# Cu_2_(OH)PO_4_/reduced graphene oxide nanocomposites for enhanced photocatalytic degradation of 2,4-dichlorophenol under infrared light irradiation†

**DOI:** 10.1039/c7ra12684k

**Published:** 2018-01-17

**Authors:** Chenyang Zhang, Zhen Du, Ruyi Zhou, Peng Xu, Xinghua Dong, Yanyan Fu, Qing Wang, Chunjian Su, Liang Yan, Zhanjun Gu

**Affiliations:** College of Mechanical and Electronic Engineering, Shandong University of Science and Technology Qingdao 266590 P. R. China suchunjian2008@163.com; CAS Key Laboratory for Biomedical Effects of Nanomaterials and Nanosafety, Institute of High Energy Physics, Chinese Academy of Sciences Beijing 100049 P. R. China yanliang@ihep.ac.cn zjgu@ihep.ac.cn; University of Chinese Academy of Sciences Beijing 101408 P. R. China; CAS Key Laboratory of Standardization and Measurement for Nanotechnology, National Center for Nanoscience and Technology Beijing 100190 P. R. China; School of Material Science and Engineering, Shandong University of Science and Technology Qingdao 266590 P. R. China; State Key Lab of Transducer Technology, Shanghai Institute of Microsystem and Information Technology, Chinese Academy of Sciences Changning Road 865 Shanghai 200050 P. R. China fuyy@mail.sim.ac.cn

## Abstract

Sparked by the growing environmental crises, photocatalytic degradation of chlorophenols with inexhaustible solar energy is expected to be converted into actual applications. Here, we report the preparation of the nanocomposite of Cu_2_(OH)PO_4_ and reduced graphene oxide (Cu_2_(OH)PO_4_/rGO) through a one-step hydrothermal method and examined its infrared-light photocatalytic activity in the degradation of 2,4-dichlorophenol (2,4-DCP). As evidenced by the absorption spectra and the degradation of 2,4-DCP, Cu_2_(OH)PO_4_/rGO exhibited enhanced infrared light-driven photocatalytic activity compared to pure Cu_2_(OH)PO_4_ and was very stable even after repeated cycling. More importantly, the introduction of hydrogen peroxide (H_2_O_2_) could combine the photocatalytic and photo-Fenton effects into one reaction system and maximize the infrared light photocatalytic efficiency. Typically, the rate constant of Cu_2_(OH)PO_4_/rGO and H_2_O_2_ was more than 6.25 times higher than that of only Cu_2_(OH)PO_4_/rGO, and almost 10 times greater than the value for pure Cu_2_(OH)PO_4_. Further, a plausible mechanism for the enhanced photocatalytic properties of Cu_2_(OH)PO_4_/rGO has been discussed. These findings may help the development of novel hybrid photocatalysts with enhanced infrared light photocatalytic activity for applications in the treatment of chlorophenol-contaminated wastewater.

## Abstract 2

Nanocomposites of Cu_2_(OH)PO_4_ and rGO exhibit the maximized infrared light photocatalytic activity for the degradation of 2,4-DCP due to the combination of the photocatalytic and photo-Fenton effects into one reaction system.

## Graphical abstract

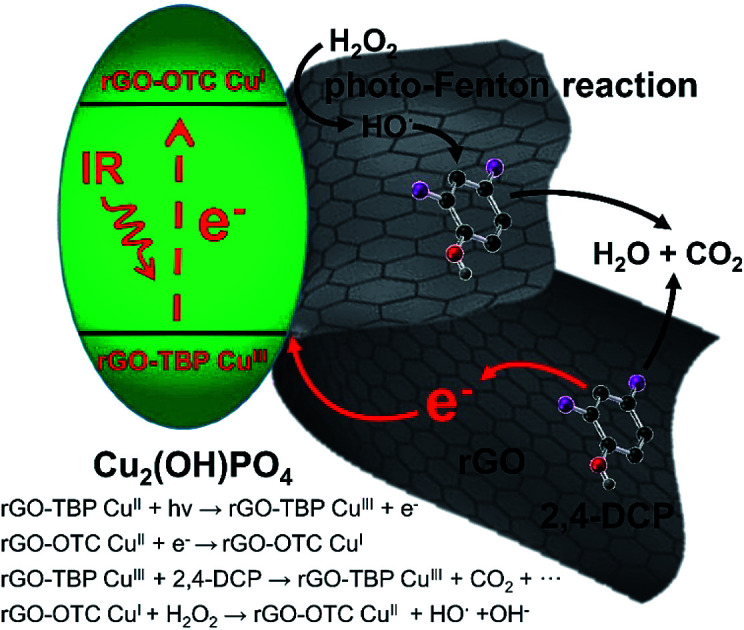

## Introduction

Pesticides are chemical substances widely used in horticulture, forestry and public health – and,^1–4^ of course, in agriculture where the unwanted pests that carry or transmit diseases can be repelled and killed.^5^ Over the past decades, tremendous problems, such as water contamination and overall ecological degradation, have been caused by the misuse and over-use of pesticides.^6–8^ For example, chlorophenols readily bio-accumulate in the human body, and subsequently cause disturbances in the structure of cellular bilayer phospholipids, finally causing carcinogenic effects.^9^ Therefore, a large number of methods have been developed to remove chlorophenols from water, including adsorption,^10^ biological degradation^11^ and electrochemical degradation.^12^ However, adsorption merely concentrates chlorophenols, but does not degrade them into less toxic compounds. Biological treatment suffers from the drawbacks of slow reaction rate and the need for strict control of suitable pH and temperature. For these reasons, an effective technique needs to be proposed for the removal of chlorophenols from different water systems.

Alternatively, semiconductor nanomaterials have been emerging as efficient photocatalysts for the degradation of chlorophenols, such as TiO_2_ in the ultraviolet range (<400 nm)^13^ and Ag_3_PO_4_ in the visible range (400–800 nm).^14^ For the optimized use of solar energy, efficient and stable photocatalysts that are capable of harvesting infrared light, which accounts for *ca.* 50% of solar energy, are required. Much effort has been under way so far to tentatively seek the efficient photocatalysts, including Bi_2_WO_6_ (ref. 15) and WS_2_,^16^ for the degradation of organic pollutants, but not chlorophenols, under infrared irradiation. The limitation of photocatalysts for pesticide photocatalytic degradation under infrared light is essentially due to the insufficient photocatalytic activity that results from charge-carrier recombination as well as the low-photon energy of infrared light, and the inhibition of charge transfer because of the mismatched band energy alignment between each other. To overcome the above limitation, graphitic carbon nitride coupled with upconversion nanoparticles can extend the activity towards the infrared region for the photodegradation of chlorophenols.^17^ However, the efficiency of this photocatalyst is rather low due to the narrow absorption band of light at 980 nm. Therefore, infrared light responsive photocatalysts for the degradation of chlorophenols are still being actively pursued.

**Scheme 1:**
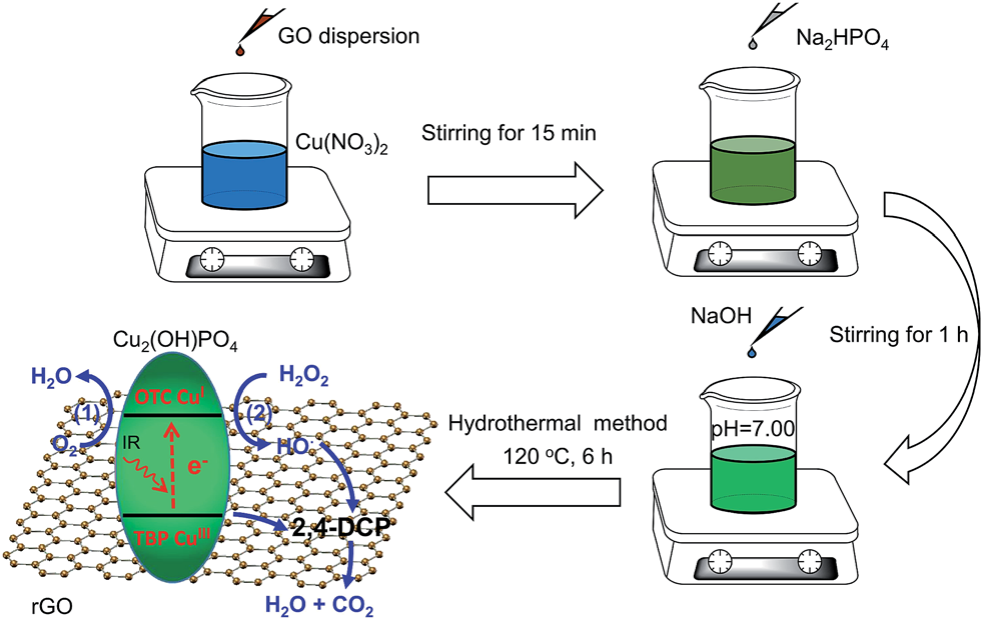
Schematic illustration of the preparation of Cu_2_(OH)PO_4_/rGO.

**Fig. 1:**
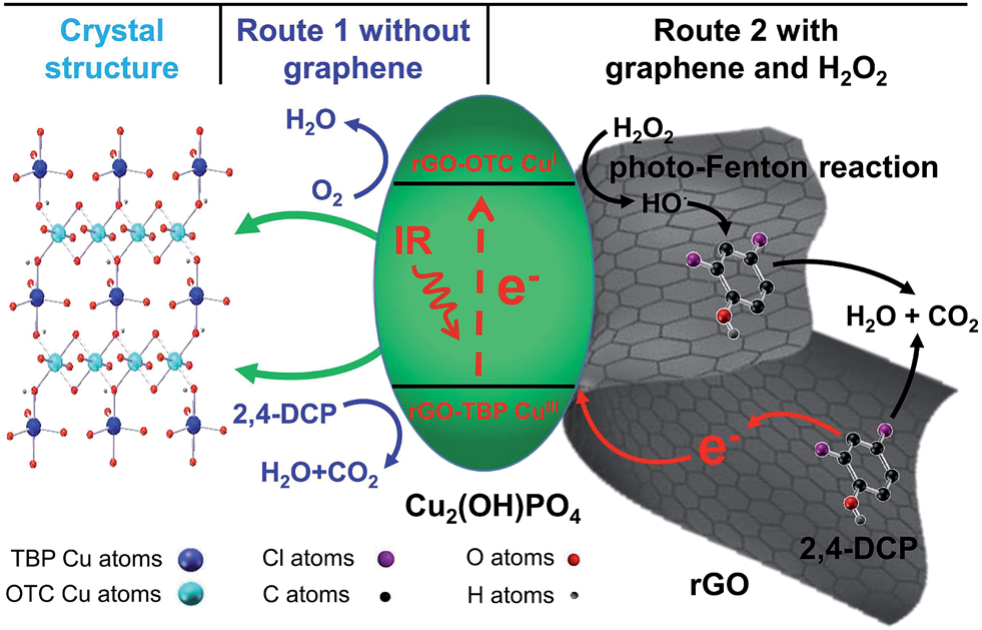
Schematic diagram for the photocatalytic mechanism of pure Cu_2_(OH)PO_4_ and Cu_2_(OH)PO_4_/rGO under infrared light irradiation.

Herein, we synthesized the infrared-light active nanocomposites composing of copper hydroxide phosphate (Cu_2_(OH)PO_4_) and reduced graphene oxide (rGO) by a one-step hydrothermal method (Cu_2_(OH)PO_4_/rGO) (Scheme 1). Cu_2_(OH)PO_4_, which consists of CuO_4_(OH)_2_ octahedron and CuO_4_(OH) trigonal bipyramid, has been considered as a promising photocatalyst for the degradation of organic pollutants under visible light.^18,19^ With the presence of the distorted polyhedrons in the crystal structure, Cu_2_(OH)PO_4_ is even responsive to infrared light and hence displays photocatalytic activity in the infrared range.^20^ In previous studies, it has been reported that the generated electrons at CuO_4_(OH) trigonal bipyramids under infrared light irradiation (forming Cu^III^ sites) can be transferred to the neighboring CuO_4_(OH)_2_ octahedra (forming Cu^I^ sites).^20,21^ Subsequently, the produced Cu^III^ sites are responsible for oxidizing chlorophenols (Fig. 1, route 1). Nevertheless, Cu_2_(OH)PO_4_, as an infrared-activated photocatalyst, suffers from the fast recombination of photogenerated electron–hole pairs. To overcome this limitation, graphene with the high-surface area and electrical conductivity should act as an avenue for driving photogenerated carriers away from the surface of Cu_2_(OH)PO_4_,^22,23^ facilitating more efficient generation of Cu^III^ sites which are applied to degrade chlorophenols and, as a result, become Cu^II^ sites (Fig. 1, route 2). Moreover, to fully use the advantage of this photocatalyst, we use hydrogen peroxide (H_2_O_2_) as an electron acceptor to react irreversibly with the produced Cu^I^ sites (that is, photo-Fenton reaction, similar to Cu^I^-induced Fenton reaction^24^) to further enhance the separation efficiency of photogenerated electron–hole pairs; while the produced Cu^I^ can effectively promote the generation of highly active hydroxyl radicals (HO˙) which are capable of oxidizing chlorophenols, and return to Cu^II^ sites. More importantly, these two processes can complete the full photocatalytic circle and be occurred repeatedly, resulting in the combination of the photocatalytic and photo-Fenton effects in one reaction system and finally maximizing the photocatalytic activity of Cu_2_(OH)PO_4_/rGO for the mineralization of chlorophenols to CO_2_ and H_2_O. As expected, our results show that, compared to pure Cu_2_(OH)PO_4_, the as-prepared Cu_2_(OH)PO_4_/rGO exhibits remarkably enhanced photocatalytic activity for the degradation of 2,4-dichlorophenol (2,4-DCP, a typical type of chlorophenols) under infrared light irradiation (>800 nm). Typically, the photocatalytic rate constant of Cu_2_(OH)PO_4_/rGO and H_2_O_2_ is almost 10 times higher than that of only Cu_2_(OH)PO_4_ under the same condition. In addition, there is no appreciable loss of photocatalytic activity after repeated cycles, and the morphology and structure of Cu_2_(OH)PO_4_/rGO remain nearly unchanged. Finally, mechanism of enhanced photocatalysis under infrared light is further proposed and discussed in detailed.

## Experimental

### Materials and chemicals

Graphite (99%) and 2,4-dichlorophenol (2,4-DCP, 97%) were purchased from Alfa Aesar. Chemical reagents including disodium hydrogen phosphate dodecahydrate (Na_2_HPO_4_·12H_2_O, 98%), copper(ii) nitrate trihydrate (Cu(NO_3_)_2_·3H_2_O, 99%) and sodium hydroxide (NaOH, 96%) were obtained from Aladdin Chemical Co. Potassium permanganate (KMnO_4_, 99%), hydrogen peroxide (H_2_O_2_, 30 wt%) and concentrated sulfuric acid (H_2_SO_4_, 98 wt%) were obtained from Beijing Chemical Co. Terephthalic acid (TA, 99%) was purchased from Sigma-Aldrich. HUVECs (human umbilical vein endothelial cells) and cell counting kit-8 (CCK-8) were acquired from Wuhan Boster Biological Technology Ltd (Wuhan, China). Dulbecco's Modified Eagle Medium (DMEM), fetal bovine serum (FBS) and Penicillin–Streptomycin Solution were purchased from Gibco (Shanghai, china). All of the reagents were analytical grade and were used without further purification. In addition, deionized water was used in the whole experimental process.

### Synthesis of nanocomposites with different mass ratios of Cu_2_(OH)PO_4_ to graphene oxide

A series of nanocomposites of Cu_2_(OH)PO_4_ and rGO with various mass ratios of graphene oxide (GO) were synthesized by a simple one-step hydrothermal method (Cu_2_(OH)PO/rGO).^20^ Briefly, 4 mL of Cu(NO_3_)_2_ solution (1.0 M) and an appropriate amount of GO dispersion (2.0 mg mL^−1^, ESI†) were mixed into 20 mL of deionized water under constantly stirring for 15 min. Then, 2 mL of Na_2_HPO_4_ solution (1.0 M) was added to the above mixture. After stirring for another 1 h, the pH value of the obtained mixture was adjusted to ∼7.00 through gradually adding NaOH aqueous solution. The resulting suspension was then transferred into a 45 mL sealed teflon-scaled autoclave and kept at 120 °C for 6 h. After naturally cooling to room temperature, the products were collected by centrifuging, washed with deionized water several times, and finally dried overnight in a freezer dryer for further use. According to the mass ratios of Cu_2_(OH)PO_4_ and GO in the original mixtures, nanocomposites were labelled as 1 : 0.001, 1 : 0.002, 1 : 0.005, 1 : 0.01, 1 : 0.02, 1 : 0.05 and 1 : 0.1, respectively.

### Characterization

Transmission electron microscopy (TEM) images were obtained on a JEM-2100 microscope at an acceleration voltage of 200 kV. Morphologies of samples were characterized using scanning electron microscopes (FE-SEM, S-4800, an acceleration voltage of 10 kV, Hitachi High-technologies, Japan) with an energy-dispersive X-ray (EDX) acceleration voltage analyzer. X-ray diffraction (XRD) patterns was obtained from a Bruker D8 Advance X-ray diffractometer (Bruker, USA) with Cu-Kα radiation (*λ* = 1.5406 Å) at a scanning rate of 10° min^−1^ and a scanning range from 10° to 90°. X-ray photoelectron spectroscopy (XPS) measurement was carried out with an ESCALab220i-XL spectrometer using a twin-anode Al-Kα X-ray source (1486.6 eV). Micro-Raman spectra were achieved from a Raman Spectroscope (Renishaw inVia plus, United Kingdom) under ambient conditions with 514 nm excitation from an argon ion laser. Ultraviolet-visible (UV-vis) data were acquired with a U-3900 spectrophotometer (Hitachi, Ltd., Japan). Ultraviolet-visible-infrared (UV-vis-IR) diffuse reflectance spectra were recorded at room temperature on an Agilent Cary 500 UV-vis-IR Spectrometer equipped with an integrating sphere using BaSO_4_ as a reference. The photoluminescence spectra were obtained using a Horiba Jobin Yvon FluoroLog3 spectrometer.

### Photocatalytic performance measurement

The photocatalytic activity of Cu_2_(OH)PO_4_/rGO was evaluated by the degradation of 2,4-DCP in the presence and absence of H_2_O_2_ under infrared light irradiation. In addition, the photocatalytic activity of Cu_2_(OH)PO_4_ and sample 1 : 0.005 to 2,4-DCP was explored under visible light irradiation. Typically, 60 mL mixture of sample 1 : 0.005 (60 mg) and 2,4-DCP (30 μg mL^−1^) was firstly stirred in the dark for 100 min to achieve an adsorption/desorption equilibrium between the photocatalyst and 2,4-DCP. Afterwards, an appropriate amount of H_2_O_2_ aqueous solution was added into the obtained mixture prior to photo-irradiation if necessary. Then, the mixture was irradiated by infrared light (>800 nm) using a 300 W xenon lamp installed an 800 nm cut-off filter, where the transmission spectrum of the cut-off filter was shown in Fig. S1.† Then, 1.5 mL of mixture was collected at varied irradiation time, centrifuged, and finally analyzed by a UV-3900 UV-vis spectrophotometer to determine the concentration of 2,4-DCP in the absence of H_2_O_2_ or by a Multi TOC Analyzer (2100, Analytik Jena AG Corporation) to detect total organic carbon (TOC) in the presence of H_2_O_2_. The photodegradation of 2,4-DCP by other Cu_2_(OH)PO/rGO were also performed under the similar condition. In addition, the stability of Cu_2_(OH)PO/rGO was studied by a separated photodegradation experiment which repeatedly re-employed the used samples for the next cycle under the identical conditions. After each photocatalytic process, Cu_2_(OH)PO/rGO was recovered by centrifugation, washed with deionized water, and then dried in the freezer dryer under vacuum for 24 h before until the subsequent reaction cycle.

### Detection of hydroxyl radical

The generation of HO˙ by nanocomposites under infrared light irradiation was evaluated using terephthalic acid (TA).^25^ The concentration of hydroxyl radical (HO˙) is determined *via* monitoring the fluorescence of 2-hydroxy terephthalic acid (TAOH, the maximum fluorescence peak at 435 nm) which is formed from the reaction of HO˙ with TA. Taking sample 1 : 0.005 as an example, five groups were obtained: group I (TA and infrared light); group II (TA, H_2_O_2_ and infrared light); group III (sample 1 : 0.005 and infrared light); group IV (TA, sample 1 : 0.005 and infrared light); group V (TA, H_2_O_2_, sample 1 : 0.005 and infrared light). The final working concentrations were 50 μg mL^−1^, 100 μM and 500 μM for sample 1 : 0.005, H_2_O_2_ and TA, respectively. After infrared light irradiation, the changes at the 435 nm fluorescence emission peak were recorded.

### Cytotoxicity assay for Cu_2_(OH)PO_4_/rGO nanocomposite

The cytotoxicity of Cu_2_(OH)PO_4_/rGO nanocomposites with 1 : 0.005 ratio to HUVECs (human umbilical vein endothelial cells) was assessed by using the CCK-8 assay. HUVECs were placed in 96-well plates at a density of 8 × 10^3^ cells per well, where HUVECs were maintained in the DMEM medium with 10% FBS, 1% penicillin/streptomycin for 24 h at 37 °C in 5% CO_2_. Then, the cells were incubated with different concentrations of sample 1 : 0.005 (5, 10, 20, 40, 60, 80, and 90 μg mL^−1^). After incubation for another 24 h, the cell medium was removed and replaced with 100 μL of fresh culture medium containing 10 μL of CCK-8 solution per well for detecting the cell viability. After 1 h of incubation, cell viability on HUVECs was assayed by measuring the absorbance at 450 nm using a microplate reader (Thermo Scientific, Multiscan MNK3).

## Results and discussion

**Fig. 2:**
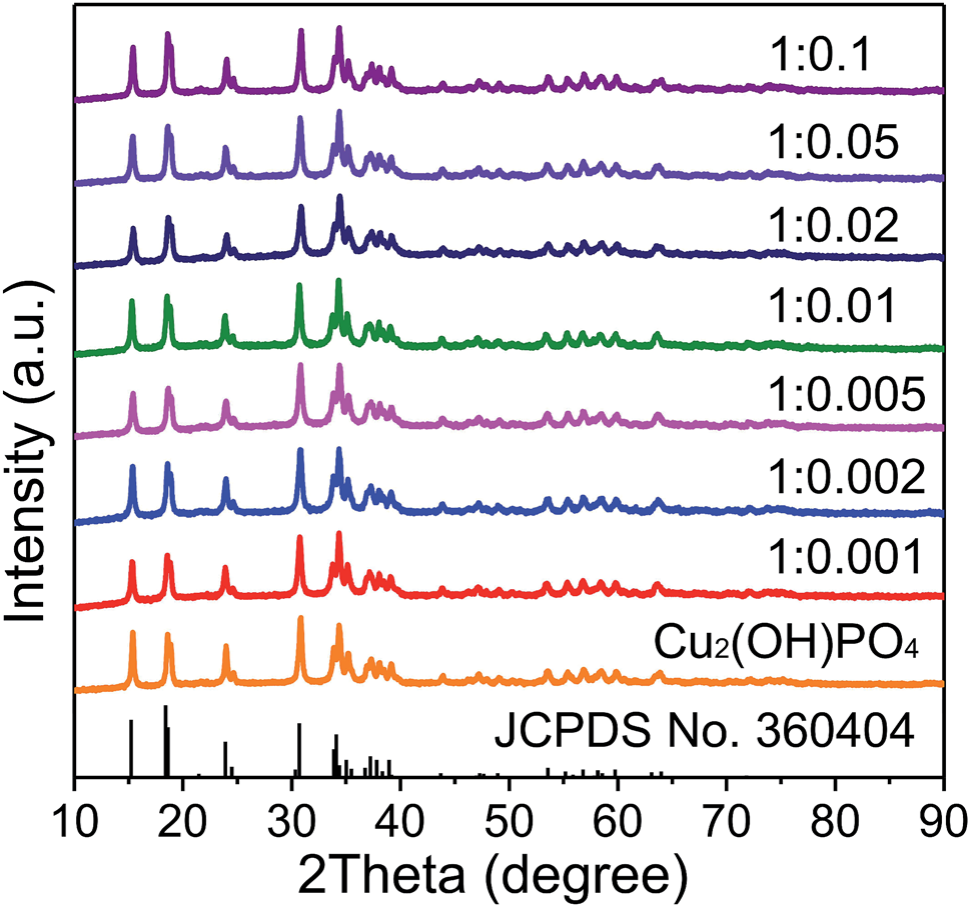
XRD patterns of pure Cu_2_(OH)PO_4_ and Cu_2_(OH)PO_4_/rGO.

XRD patterns of pure Cu_2_(OH)PO_4_ and Cu_2_(OH)PO_4_/rGO with different mass ratios of Cu_2_(OH)PO_4_ and GO nanosheets with the thickness of ∼1.2 nm (Fig. S2†) are displayed and compared in Fig. 2. According to JCPDS card no. 360404, all the diffraction peaks are associated with the orthorhombic phase of Cu_2_(OH)PO_4_.^26^ Moreover, it is clear that the position of the diffraction peaks of Cu_2_(OH)PO_4_/rGO basically keep unchanged, which indicates that the crystalline structure of Cu_2_(OH)PO_4_ is not affected after the introduction of GO. Additionally, no stacking-related (002) diffraction peaks of graphene (at ∼26° for graphite and ∼13° for graphite oxide) are detected, suggesting that the dispersion of graphene is probably close to the single-sheet level in all the nanocomposites.^27^

**Fig. 3:**
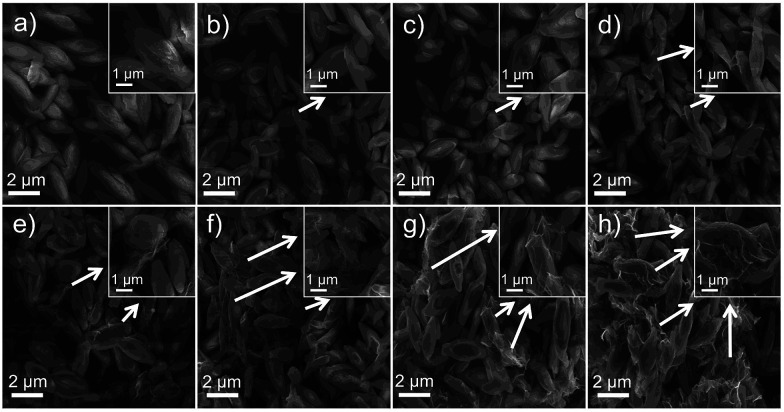
SEM images of pure Cu_2_(OH)PO_4_ (a) and Cu_2_(OH)PO_4_/rGO nanocomposites with 1 : 0.001 (b), 1 : 0.002 (c), 1 : 0.005 (d), 1 : 0.01 (e),1 : 0.02 (f), 1 : 0.05 (g) and 1 : 0.1 (h) ratios. Inset: enlarged SEM images of pure Cu_2_(OH)PO_4_ (a) and Cu_2_(OH)PO_4_/rGO.

The morphology of pure Cu_2_(OH)PO_4_ and Cu_2_(OH)PO_4_/rGO were characterized by SEM and TEM measurement. As shown in Fig. 3, pure Cu_2_(OH)PO_4_ are ellipsoid-shaped with an average length of 3–4 μm and an aspect ratio of ∼3, and there are ravines on their surface that are short and narrow. In contrast, with the addition of GO, Cu_2_(OH)PO_4_ are tightly encapsulated by (rGO) nanosheets, which is consistent with the TEM images shown in Fig. S3.† This indicates the presence of strong van der Waals force between graphene and Cu_2_(OH)PO_4_.^28^ In addition, on increasing the mass ratio of GO, more Cu_2_(OH)PO_4_ is encapsulated by graphene, exhibiting more obviously crinkled and rough textures on the surface of Cu_2_(OH)PO_4_/rGO. Remarkably, graphene nanosheets not only are adsorbed onto the surface of Cu_2_(OH)PO_4_/rGO tightly, but also are connected or even overlapped between the adjacent microcrystals, building interconnected conductive pathways for electron transfer.

**Fig. 4:**
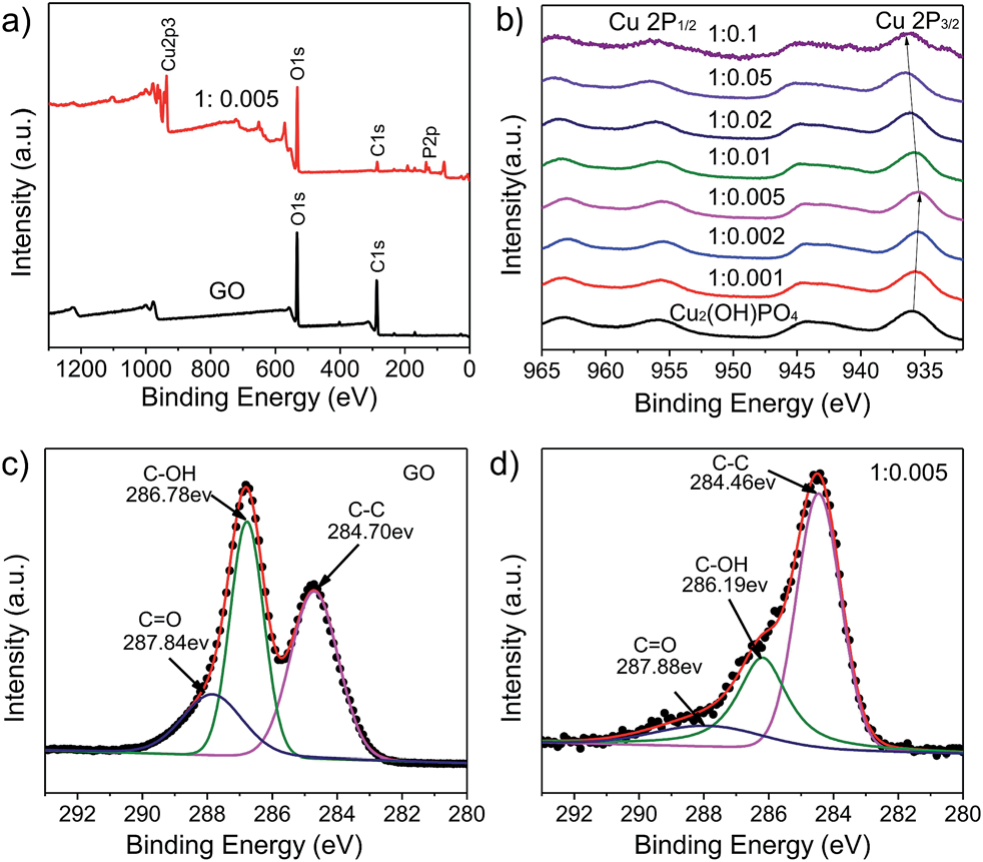
(a) Full-scale XPS spectra of GO and sample 1 : 0.005. (b) Cu 2p XPS spectra of pure Cu_2_(OH)PO_4_ and Cu_2_(OH)PO_4_/rGO. (c) C 1s XPS spectrum of GO. (d) C 1s XPS spectrum of sample 1 : 0.005.

The structural and chemical information of Cu_2_(OH)PO_4_/rGO was further studied using Raman spectroscopy and XPS measurement. Raman spectra of GO, rGO, pure Cu_2_(OH)PO_4_ and Cu_2_(OH)PO_4_/rGO are displayed in Fig. S4a.† From Raman spectra of pure Cu_2_(OH)PO_4_ and Cu_2_(OH)PO_4_/rGO, vibration peak at ∼972.5 cm^−1^ is the characteristics of Cu_2_(OH)PO_4_. Raman spectra of rGO exhibits two characteristic peaks corresponding to D band at around 1350.4 cm^−1^ (involving the disorder and defect) and G band at about 1599.6 cm^−1^ (involving first order scattering of the tangential stretching phonon mode), respectively.^29,30^ In comparison to rGO, Cu_2_(OH)PO_4_/rGO also shows the typical features of graphene with the presence of D band and G band, indicating the successful combination of Cu_2_(OH)PO_4_ with rGO. Interestingly, it can be obviously found from Fig. S4b† that, on progressively increasing the proportion of GO, there is significant red-shift in the G band; while the D band firstly blue-shifts to 1361.14 cm^−1^ from samples 1 : 0.001 to 1 : 0.005, and then red-shifts to 1351.95 cm^−1^ from samples 1 : 0.005 to 1 : 0.1. This indicates the presence of van der Waals interaction alone with charge transfer between rGO and Cu_2_(OH)PO_4_, and the interaction is the strongest for sample 1 : 0.005.^31^ Moreover, the D/G intensity ratios of Cu_2_(OH)PO_4_/rGO are larger than that of GO (*I*_D_/*I*_G_ = 0.806), which suggests a decrease in the average size of the sp^2^ domains upon reduction of GO, as well as an increase of edge planes and the degree of disorder.^32^ The full-scale XPS spectra of Cu_2_(OH)PO_4_/rGO shown in Fig. 4a and S5† reveal the presence of P, O, and Cu elements, which is consistent with the EDS results shown in Fig. S6.† For the XPS spectra of pure Cu_2_(OH)PO_4_, two main peaks are observed at about 936.61 and 955.86 eV, which can be attributed to Cu 2p_3/2_ and Cu 2p_1/2_ of copper ions, respectively (Fig. 4b).^33^ More importantly, the peak position of Cu 2p_3/2_ in the Cu_2_(OH)PO_4_/rGO firstly decreases from 935.76 eV to 935.46 eV as the mass ratio increases from 1 : 0.001 to 1 : 0.005, and then increases to 936.56 eV on further increasing the proportion of GO. This may be due to the screening effect *via* charge transfer between rGO and pure Cu_2_(OH)PO_4_.^34^ With low mass ratio of GO, the electron of rGO can effectively transfer to pure Cu_2_(OH)PO_4_, causing the shift of both Cu 2p_3/2_ and Cu 2p_1/2_ towards lower binding energy; whereas, above the mass ratio of 1 : 0.005, rGO begins to aggregate, thereby leading to inhibit electron transfer. Therefore, we predict that sample 1 : 0.005 may be the optimal mass ratio for the photocatalytic degradation of chlorophenols. Furthermore, the C 1s XPS spectra of GO and Cu_2_(OH)PO_4_/rGO are shown in the Fig. 4c and d. For GO, three different peaks located at 284.70, 286.78 and 287.84 eV can be obtained, corresponding to C=C, C–OH, and C=O bonds, respectively.^35,36^ There is also three peaks at the same position for Cu_2_(OH)PO_4_/rGO (ESI, Fig. S7†); while the peak areas of C–OH and C=O significantly decrease compared to GO, suggesting the oxygen functional groups are removed during the hydrothermal treatment. This is good agreement with the result obtained by the Raman spectroscopy. These demonstrate that graphene is successfully combined with Cu_2_(OH)PO_4_ and simultaneously is reduced effectively during the hydrothermal reaction. Therefore, this structure of Cu_2_(OH)PO_4_/rGO can enhance their photocatalytic activity under infrared light *via* promoting charge separation of photocarriers.^37,38^

The optical properties of pure Cu_2_(OH)PO_4_ and Cu_2_(OH)PO_4_/rGO are investigated by UV-vis-NIR diffuse reflectance spectroscopy (DRS). As shown in Fig. S8a,† pure Cu_2_(OH)PO_4_ displays a strong absorption in the infrared region, which can be further fitted with four Gaussian peaks centered at 662, 777, 965 and 1237 nm (1.55 eV, 1.32 eV, 1.06 eV and 0.88 eV, respectively, Fig. S8b†). According to the previous report, the absorption peak at ∼777 nm is mainly attributed to the ^2^E_g_–B_1g_ transition for Cu^II^ sites that exists in axially elongated CuO_4_(OH)_2_ octahedra, and the absorption peaks located at ∼662, 965, and 1237 nm are largely associated with d–d transitions for Cu^II^ sites that exist in axially compressed CuO_4_(OH) trigonal bipyramids.^20,39,40^ On the introduction of GO into Cu_2_(OH)PO_4_, Cu_2_(OH)PO_4_/rGO exhibits enhanced optical absorption in infrared region over pure Cu_2_(OH)PO_4_, with red-shift in the maximum absorption peak. (Fig. S8c, d and S9†) Note that the color of Cu_2_(OH)PO_4_/rGO becomes much darker with the increasing mass ratio of GO. Remarkably, when mass ratio of GO reaches up to 1 : 0.01, the absorption peak in the infrared region gradually decreases because that the excessive graphene is capable of shielding the infrared light and inhibits the photo absorption by Cu_2_(OH)PO_4_.^41,42^

**Fig. 5:**
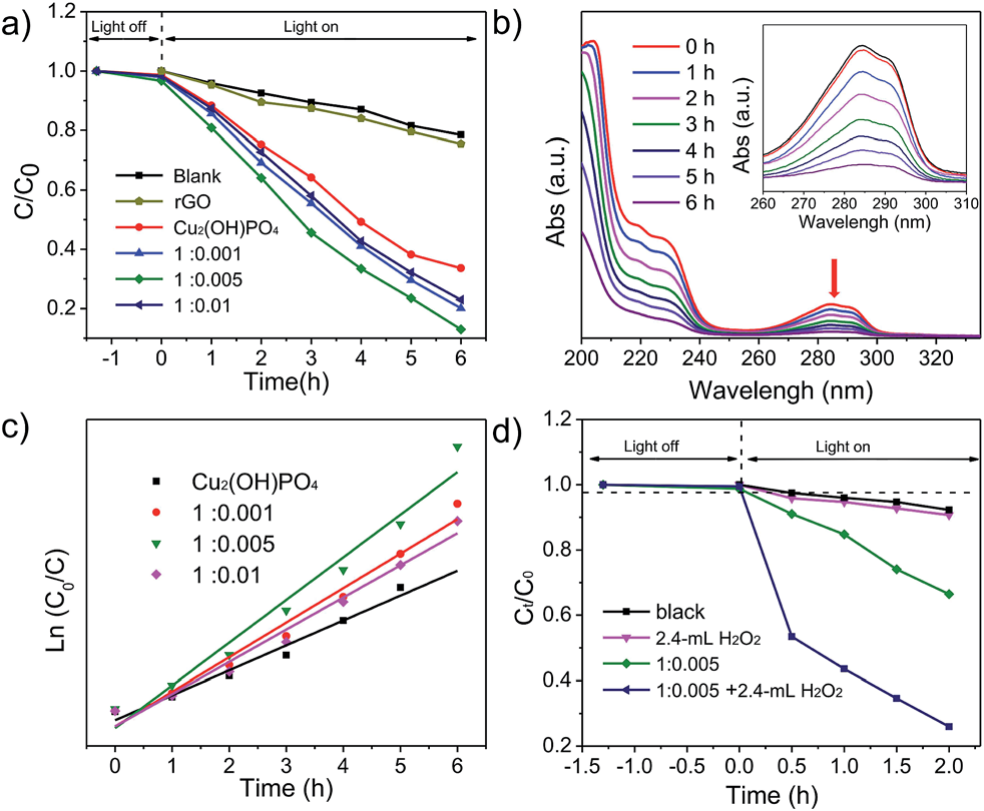
(a) Photodegradation of 2,4-DCP over pure Cu_2_(OH)PO_4_ and Cu_2_(OH)PO_4_/rGO nanocomposites with 1 : 0.001, 1 : 0.005 and 1 : 0.01 ratios with infrared light irradiation at 20–25 °C. (b) UV-vis absorption spectra the aqueous solution of 2,4-DCP with the irradiation time. Inset: enlarged spectra ranges from 260 nm to 310 nm. (c) Plots of ln(*C*_0_/*C*) *versus* time for pure Cu_2_(OH)PO_4_ and Cu_2_(OH)PO_4_/rGO nanocomposites with 1 : 0.001, 1 : 0.005 and 1 : 0.01 ratios. (d) Photodegradation efficiency of 2,4-DCP for sample 1 : 0.005 in the presence of 2.4 mL of H_2_O_2_ under infrared light irradiation.

To evaluate the photocatalytic properties of Cu_2_(OH)PO_4_/rGO under infrared light (*λ* > 800 nm), 2,4-DCP, which is fairly stable under solar light irradiation, is used as a model pollutant. To minimize the loss of 2,4-DCP by evaporation, the temperature of the solution was maintained at 20–25 °C during infrared light irradiation. Fig. 5a and S10a† shows time profiles of *C*_*t*_/*C*_0_ under infrared light irradiation in the presence of Cu_2_(OH)PO_4_/rGO, where *C*_*t*_ is the concentration of 2,4-DCP at the irradiation time of *t* and *C*_0_ is the concentration in the adsorption equilibrium of the photocatalysts before photo-irradiation. The results show that almost no photolysis is observed without photocatalysts after 6 h of infrared light irradiation. 2,4-DCP is slightly degraded in the presence of Cu_2_(OH)PO_4_; while the degradation is remarkably accelerated with Cu_2_(OH)PO_4_/rGO. On progressively increasing the proportion of GO, the degradation rate initially increases for Cu_2_(OH)PO_4_/rGO, and then declines. Among all the nanocomposites, sample 1 : 0.005 shows the highest degradation rate (87.1%) after irradiation with infrared light for 6 h, which is rather higher than that of pure Cu_2_(OH)PO_4_ (66.4%). This enhancement clearly indicates effective recombination suppression, which can be attributed to the van der Waal heterojunction between Cu_2_(OH)PO_4_ and rGO. This is consistent with the results of fluorescence spectra of pure Cu_2_(OH)PO_4_, 1 : 0.001, 1 : 0.005 and 1 : 0.1, which is shown in Fig. S11.† However, from samples 1 : 0.01 to 1 : 0.1, the degradation rate decreases to 78.5% after 6 h of infrared light irradiation. This is largely attributed to the fact that the excessive graphene shields Cu_2_(OH)PO_4_, preventing absorption and blocking electron transfer between Cu_2_(OH)PO_4_ and graphene, as demonstrated by the results of XPS, Raman spectrum and DRS measurements. It is worth noting that these values are still higher than that of pure Cu_2_(OH)PO_4_. Fig. 5b shows the evaluation of the absorption spectra of 2,4-DCP with reaction time in the presence of sample 1 : 0.005.

The pseudo-first order kinetic model is then used for the determination of the photocatalytic degradation rate constant (*k*, h^−1^) which is expressed by eqn (1):^43,44^

1 ln(*C*_0_/*C*_*t*_) = *kt*,

Fig. 5c and S10b† depict the ln(*C*_0_/*C*_*t*_) *versus t* for pure Cu_2_(OH)PO_4_ and Cu_2_(OH)PO_4_/rGO. Pure Cu_2_(OH)PO_4_ presents an apparent photocatalytic rate constant of 0.190 h^−1^ under the irradiation of infrared light. However, the photocatalytic rate constants of Cu_2_(OH)PO_4_/rGO for 2,4-DCP are higher than that of pure Cu_2_(OH)PO_4_ under the same condition. Among these Cu_2_(OH)PO_4_/rGO with various mass ratios, sample 1 : 0.005 shows the highest photocatalytic rate constant, which is 1.72 times than that of pure Cu_2_(OH)PO_4_. The photocatalytic rate constant of other samples is summarized in Table S1.†

The above results clearly demonstrate that graphene plays a significant role in the enhanced infrared light photocatalytic activity. A tentative photocatalytic mechanism of Cu_2_(OH)PO_4_/rGO for degradation of 2,4-DCP under infrared light irradiation is proposed and schematically illustrated in Fig. 1. Similar to pure Cu_2_(OH)PO_4_,^20^ copper atoms of Cu_2_(OH)PO_4_/rGO have two nonequivalent crystallographic sites, including octahedral sites (rGO-OCT Cu^II^) and trigonal bipyramidal sites (rGO-TBP Cu^II^). In this system, the distorted polyhedrons can lead to a net dipole moment in such units, facilitating electron transfer from rGO-TBP Cu^II^ to neighboring rGO-OCT Cu^II^. Benefiting from the strong absorption in infrared region, Cu_2_(OH)PO_4_/rGO can be excited by infrared light and therefore generate electrons (forming rGO-OCT Cu^I^ sites at the CuO_4_(OH)_2_ octahedra) and holes (forming rGO-TBP Cu^III^ sites at the CuO_4_(OH) trigonal bipyramids). Then, by taking advantage of the high-specific area surface and electrical conductivity, graphene nanosheets can provide more sites to absorb 2,4-DCP and promote photogenerated holes transfer from rGO-TBP Cu^III^ to 2,4-DCP, which is responsible for degrading 2,4-DCP. In parallel, rGO-OCT Cu^I^ may drive the half-reaction of oxygen reduction. The main processes in the photocatalytic degradation of 2,4-DCP could be summarized as follows:

2 rGO-TBP Cu^II^ + hv → rGO-TBP Cu^III^ + e^−^

3 rGO-OCT Cu^II^ + e^−^ → rGO-OCT Cu^I^

4 rGO-TBP Cu^III^ + 2,4-DCP → rGO-TBP Cu^II^ + H_2_O + CO_2_ + H^+^ + Cl^−^

5 rGO-OCT Cu^I^ + H^+^ + O_2_ → rGO-OCT Cu^II^ + H_2_O

Furthermore, the introduction of H_2_O_2_ as a sacrificial electron acceptor can maximize the photocatalytic efficiency for the degradation of 2,4-DCP with Cu_2_(OH)PO_4_/rGO through the combination of the photocatalytic and photo-Fenton effects into one reaction system. Taking sample 1 : 0.005 as an example, it is found that the photocatalytic activity is gradually improved on the increase of the amount of H_2_O_2_ (Fig. S12†). As shown in Fig. 5d, when 2.4 mL H_2_O_2_ is added, the photodegradation efficiency of 2,4-DCP increases from 33.6% to 74.1% after 2 h of infrared light irradiation. According to the kinetic curves in Fig. S13,† the photocatalytic rate constant of sample 1 : 0.005 and 2.4 mL H_2_O_2_ is more than 6.25 times higher than the corresponding value for only sample 1 : 0.005, and almost 10 times greater than the value for pure Cu_2_(OH)PO_4_. These results confirm that the addition of H_2_O_2_ as a sacrificial acceptor can remarkably enhance the photocatalytic efficiency of Cu_2_(OH)PO_4_/rGO for the degradation of 2,4-DCP under infrared light irradiation. In addition, it is found from Fig. S14† that the photocatalytic properties of sample 1 : 0.005 is not much better than pure Cu_2_(OH)PO_4_ under visible light irradiation. At the same time, sample 1 : 0.005 shows the higher degradation rate (87.1%) after irradiation with infrared light for 6 h relative to the degradation rate (54.5%) with visible light irradiation, which it is possible that the redox potential of 2,4-DCP is not match the band position of Cu_2_(OH)PO_4_ corresponding to visible light.

The mechanism of photocatalytic degradation of 2,4-DCP by Cu_2_(OH)PO_4_/rGO with H_2_O_2_ as the sacrificial electron acceptor is proposed as follows:

6 rGO-OCT Cu^I^ + H_2_O_2_ → rGO-OCT Cu^II^ + HO˙ + OH^−^

7 H^+^ + OH^−^ → H_2_O

8 2,4-DCP + HO˙ → H_2_O + CO_2_ + H^+^ + Cl^−^

It is generally accepted that H_2_O_2_ is a better sacrificial electron acceptor than O_2_.^45^ Therefore, in the presence of H_2_O_2_, rGO-OCT Cu^I^ produced under infrared light irradiation can react irreversibly with H_2_O_2_ to (a) further improve the electron–hole separation and (b) the effective generation of powerful reactive species HO˙ radicals *via* the photo-Fenton reaction, which can further oxidize 2,4-DCP during the photocatalytic degradation reaction,^46^ finally returning to Cu^II^ sites. In addition to reaction (4), these two processes complete the full photocatalytic circle and are occurred repeatedly, finally maximizing the photocatalytic efficiency for the mineralization of 2,4-DCP to CO_2_ and H_2_O. This is the unique advantage of these Cu_2_(OH)PO_4_/rGO as a novel infrared-light-active photocatalyst. As shown in Fig. S15,† it can be easily seen that sample 1 : 0.005 is capable of catalyzing H_2_O_2_ efficiently to generate HO˙ only when irradiated with infrared light. The amount of the generated HO˙ for sample 1 : 0.005 under infrared light irradiation is over 7 times than the control groups. Remarkably, there is no generation of HO˙ without the irradiation of infrared light. These demonstrate that the infrared light photocatalytic activity is further significantly enhanced when the photocatalytic and photo-Fenton effects are combined in one reaction system.

**Fig. 6:**
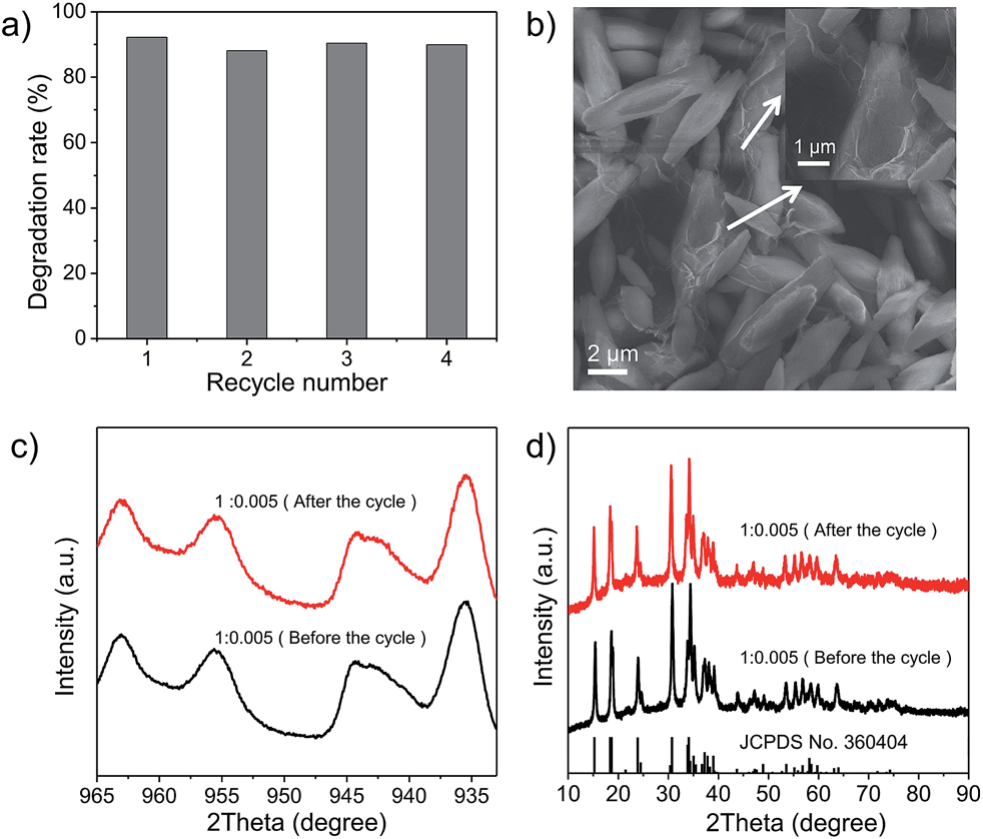
(a) Cycles of the photocatalytic degradation of 2,4-DCP in the presence of sample 1 : 0.005. (b) SEM image of sample 1 : 0.005 after the photocatalytic reaction cycles. Inset: enlarged SEM image. (c and d) The Cu 2p XPS spectra and XRD patterns of sample 1 : 0.005 after the photocatalytic reaction cycles.

Finally, the stability of Cu_2_(OH)PO_4_/rGO was evaluated using cyclic experiments. Fig. 6a shows the degradation of 2,4-DCP for four runs of reactions. The photocatalytic efficiency of sample 1 : 0.005 does not decrease even after several cycles. This indicates that sample 1 : 0.005 can be efficiently recycled and reused for repeated cycles without appreciable loss of activity. SEM images and XPS spectra of sample 1 : 0.005 before the photocatalytic reaction and after the photocatalytic reaction cycles are presented in Fig. 6b and c, respectively. No differences have been found, either in the morphology of sample 1 : 0.005 or in their chemical structure after photocatalytic degradation. Furthermore, XRD patterns shown in Fig. 6d indicate that the crystal structures of sample 1 : 0.005 remained the same after repeated photocatalytic cycling. At the same time, SEM images, XPS spectra and XRD patterns shown in Fig. S16–S18,† respectively arrested that other Cu_2_(OH)PO_4_/rGO also possess nice photocatalytic stability. In addition, in order to evaluate the biosecurity of Cu_2_(OH)PO_4_/rGO nanocomposites, the cytotoxicity of sample 1 : 0.005 was assessed to HUVECs (human umbilical vein endothelial cells) by the CCK-8 assay. It can be seen from Fig. S19† that no obvious cytotoxicity is induced in human umbilical vein endothelial cells, even at high concentrations up to 90 μg mL^−1^, which exhibits Cu_2_(OH)PO_4_/rGO nanocomposites have good biosecurity.

## Conclusion

In summary, Cu_2_(OH)PO_4_/rGO nanocomposites with different mass ratios of GO are successfully synthesized through a one-step hydrothermal method. By coupling with graphene, the photocatalytic activity of pure Cu_2_(OH)PO_4_ is remarkably enhanced under infrared light irradiation. With an optimal mass ratio of 0.5 wt% graphene oxide, the highest rate of 2,4-DCP degradation is achieved due to the effective hybridization between Cu_2_(OH)PO_4_. Moreover, the introduction of H_2_O_2_ as the sacrificial electron acceptor can maximize the infrared light photocatalytic activity *via* the combination of the photocatalytic and photo-Fenton effects into one reaction system. In this system, the reaction of H_2_O_2_ with rGO-OCT Cu^I^ sites, as well as the coupling of graphene with Cu_2_(OH)PO_4_, can improve the separation and transportation of photogenerated electrons and holes; while the generated HO˙ *via* the photo-Fenton reaction can further oxidize 2,4-DCP. These are the unique advantages of Cu_2_(OH)PO_4_/rGO as a novel infrared-light-active photocatalyst. Typically, the photocatalytic rate constant of Cu_2_(OH)PO_4_/rGO and H_2_O_2_ is ∼6.25 times higher than the corresponding value for only Cu_2_(OH)PO_4_/rGO, and ∼10 times greater than the value for pure Cu_2_(OH)PO_4_. Moreover, Cu_2_(OH)PO_4_/rGO is very stable after many photocatalytic cycles and can be reused without significant loss of photocatalytic activity. In addition, a possible decomposition mechanism for the degradation of 2,4-DCP is further proposed. This work may help the development of a new strategy to search stable and effective photocatalysts with high infrared light photocatalytic activity and bring the promise to fulfilment of actual applications in the treatment of non-biodegradable chlorophenols with lower costs and non-secondary pollution to the environment.

## Conflicts of interest

There are no conflicts to declare.

## Supplementary Material

RA-008-C7RA12684K-s001
