# Supplementary material for: Cu2(OH)PO4/reduced graphene oxide nanocomposites for enhanced photocatalytic degradation of 2,4-dichlorophenol under infrared light irradiation
Source: RSC Adv. 2018 Jan 17;8(7):3611–8. doi: 10.1039/c7ra12684k (PMC9077714; doi:10.1039/c7ra12684k)
Supplement: RA-008-C7RA12684K-s001 [file RA-008-C7RA12684K-s001.pdf]

Electronic Supplementary Material (ESI) for RSC Advances.
This journal is © The Royal Society of Chemistry 2018

Supporting Information

Cu2(OH)PO4/reduced graphene oxide nanocomposites for enhanced photocatalytic degradation of 2,4-dichlorophenol under infrared light irradiation

Chenyang Zhang,[a,b] *Zhen Du,*[b] *Ruyi Zhou,*[b] *Peng Xu,*[d] *Xinghua Dong,*[b,c] *Yanyan Fu,*[*f] *Qing Wang,*[e] *Chunjian Su,*[*a] *Liang Yan,*[*b,c] *Zhanjun Gu*[*b,c]

[a] College of Mechanical and Electronic Engineering, Shandong University of Science and Technology, Qingdao 266590, P. R. China

[b] CAS Key Laboratory for Biomedical Effects of Nanomaterials and Nanosafety, Institute of High Energy Physics, Chinese Academy of Sciences, Beijing 100049, P. R. China

[c] University of Chinese Academy of Sciences, Beijing 101408, P. R. China

[d] CAS Key Laboratory of Standardization and Measurement for Nanotechnology, National Center for Nanoscience and Technology, Beijing 100190, P. R. China.

[e] School of material science and engineering, Shandong University of Science and Technology, Qingdao 266590, P. R. China

[f] State Key Lab of Transducer Technology, Shanghai Institute of Microsystem and Information Technology, Chinese Academy of Sciences, Changning Road 865, Shanghai 200050, China

KEYWORDS: $Cu_2(OH)PO_4$; graphene; 2,4-dichlorophenol; infrared light; photocatalysis

Synthesis of Graphene oxide

Graphene oxide were synthesized from powdered graphite via a modified Hummers method.[1-2] Typically, powdered graphite (2.0000 g) was added to 38 mL of H_2SO_4 (98 wt%) with stirring for 20 min in an ice-bath. Then $KMnO_4$ (4.0000 g) was slowly added to the above suspension under vigorous stirring. After another vigorously stirring at 37 ℃ for 2 h, 12.5 mL of H_2O_2 solution (30 wt%) was added to the above solution, and then the obtained suspension was diluted by adding 195 mL of deionized water under stirring. The resulting dispersion was then transferred into a dialysis membrane to remove the remaining metal species. Finally, the obtained product was diluted into 2.0 mg mL^{-1} and then sonicated for 1 h to obtain graphene oxide dispersion.

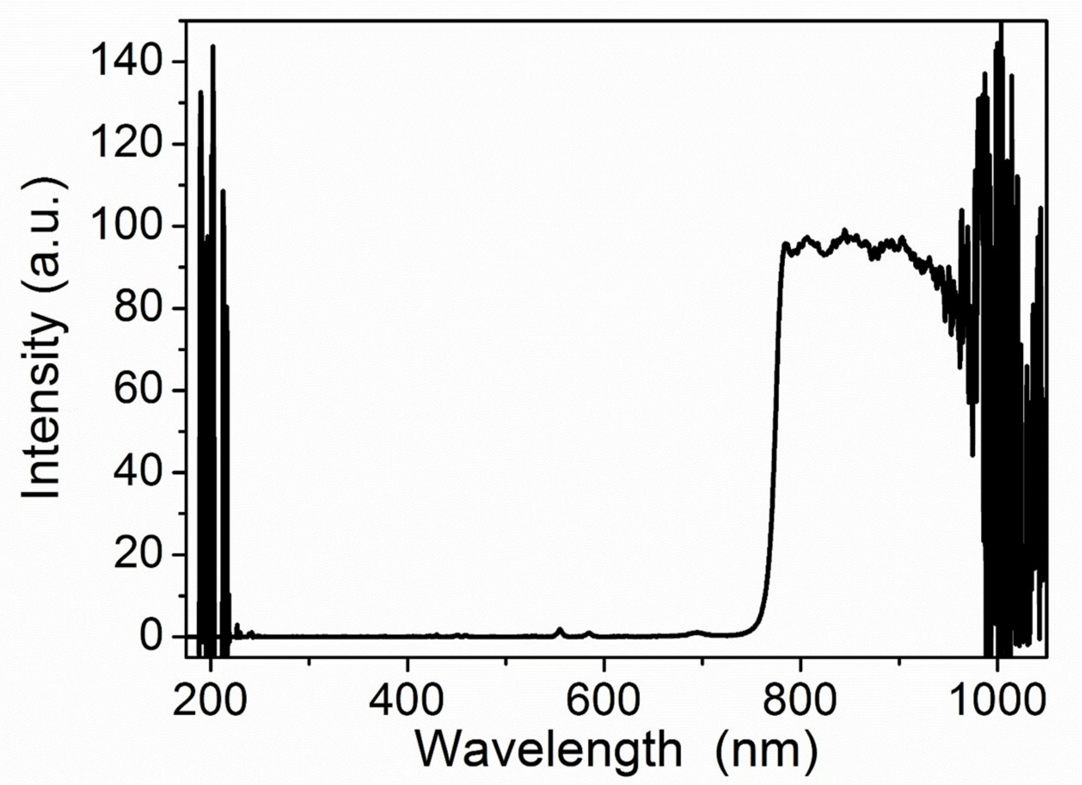

Fig. S1 Transmission spectrum of the 300-W xenon lamp installed an 800-nm cut-off filter.

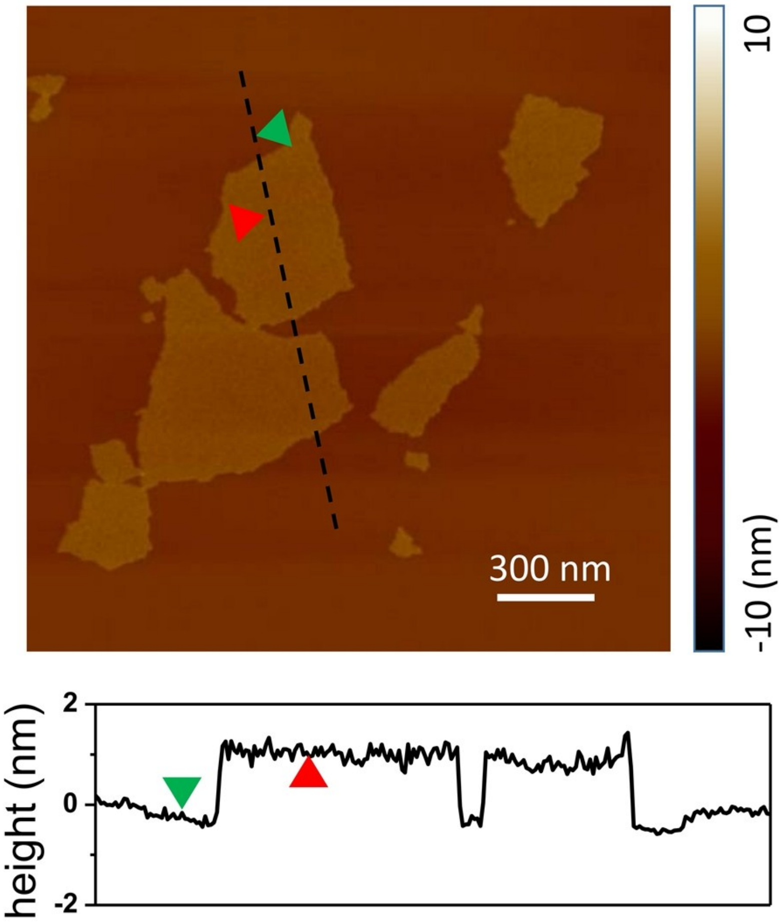

Fig. S2 AFM image of the as-prepared GO, where the AFM analysis showed that the thickness of the GO nanosheets was ca. 1.2 nm.

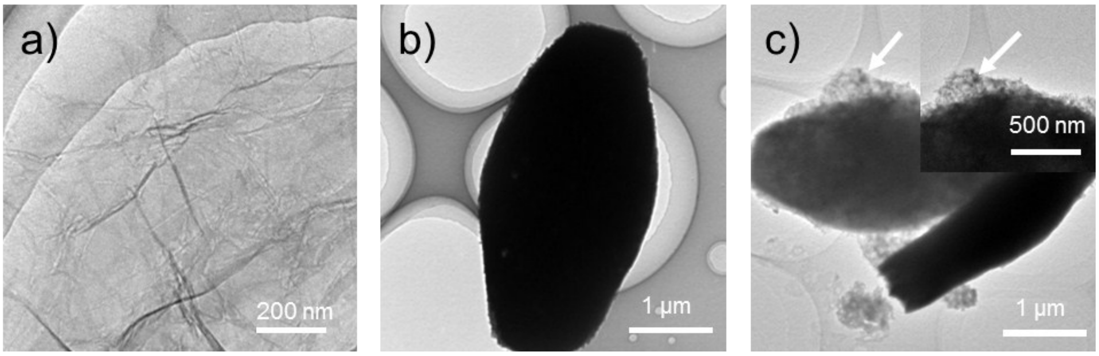

Fig. S3 TEM images of a) GO, b) pure $Cu_2(OH)PO_4$ and c) $Cu_2(OH)PO_4$/rGO nanocomposites with 1:0.005 rations.

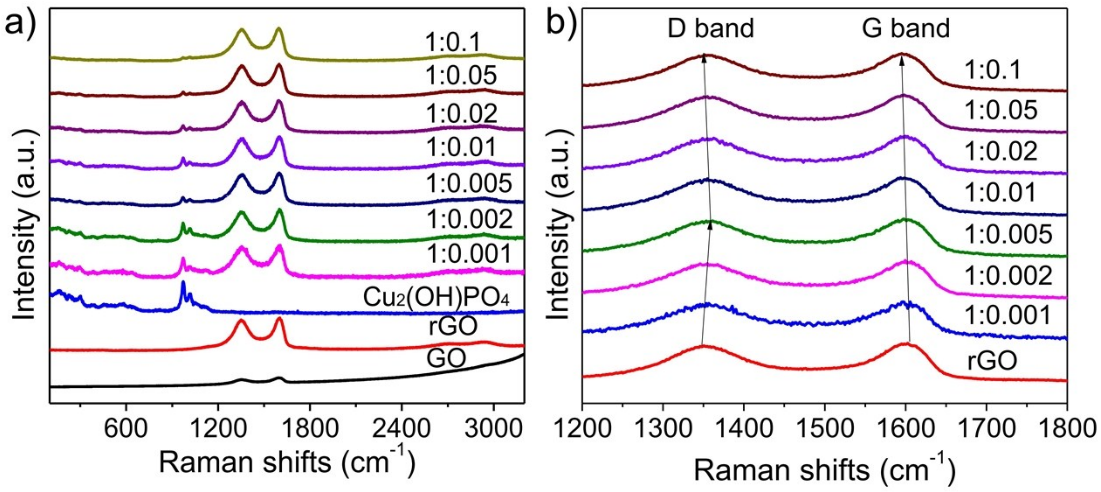

Fig. S4 (a) Raman spectra of GO, rGO, pure $Cu_2(OH)PO_4$ and $Cu_2(OH)PO_4$/rGO nanocomposites. (b) Enlarged Raman spectra of $Cu_2(OH)PO_4$/rGO nanocomposites ranges from 1200 to 1800 cm^{-1}.

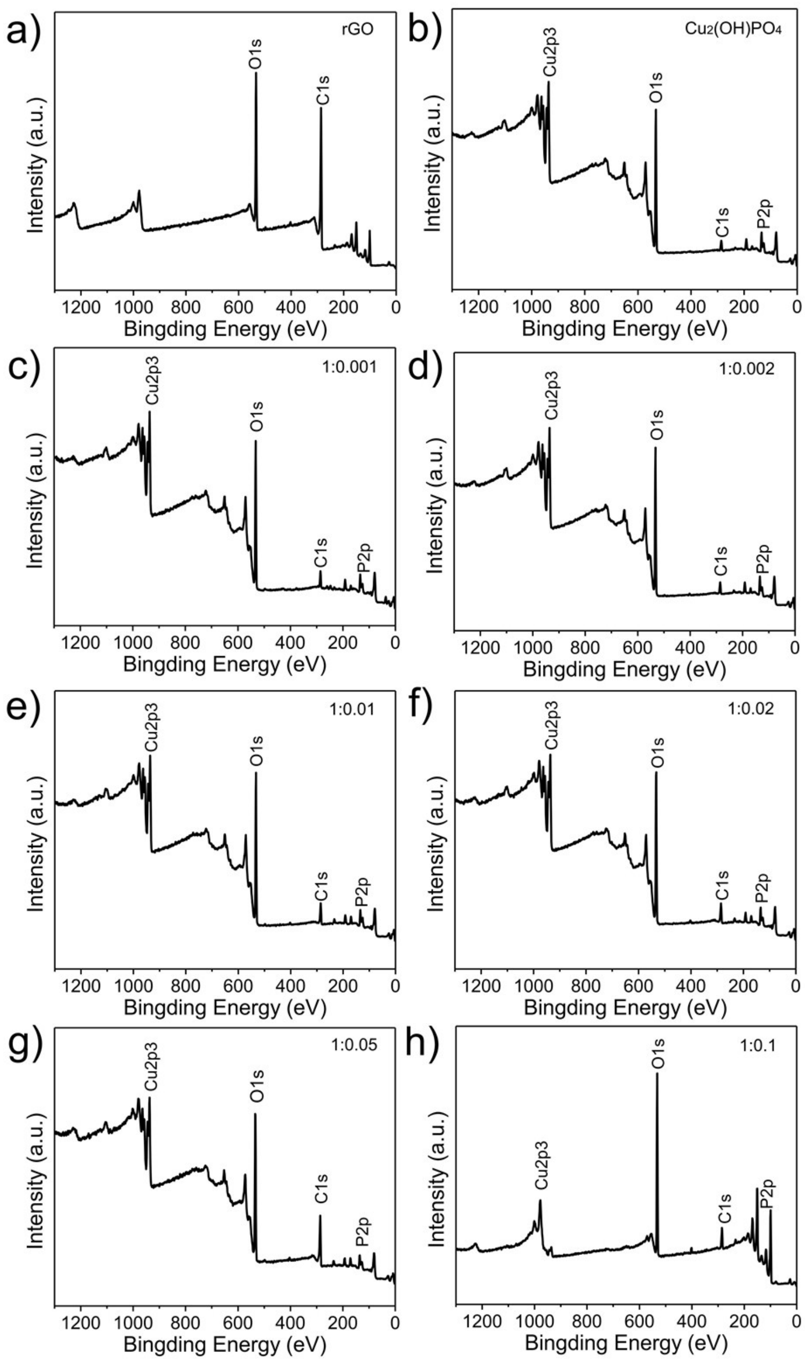

Fig. S5 Full-scale XPS spectra of rGO (a), pure $Cu_2(OH)PO_4$ (b), sample 1:0.001 (c), sample 1:0.002 (d), sample 1:0.01 (e), sample 1:0.02 (f), sample 1:0.05 (g) and sample 1:0.1 (h).

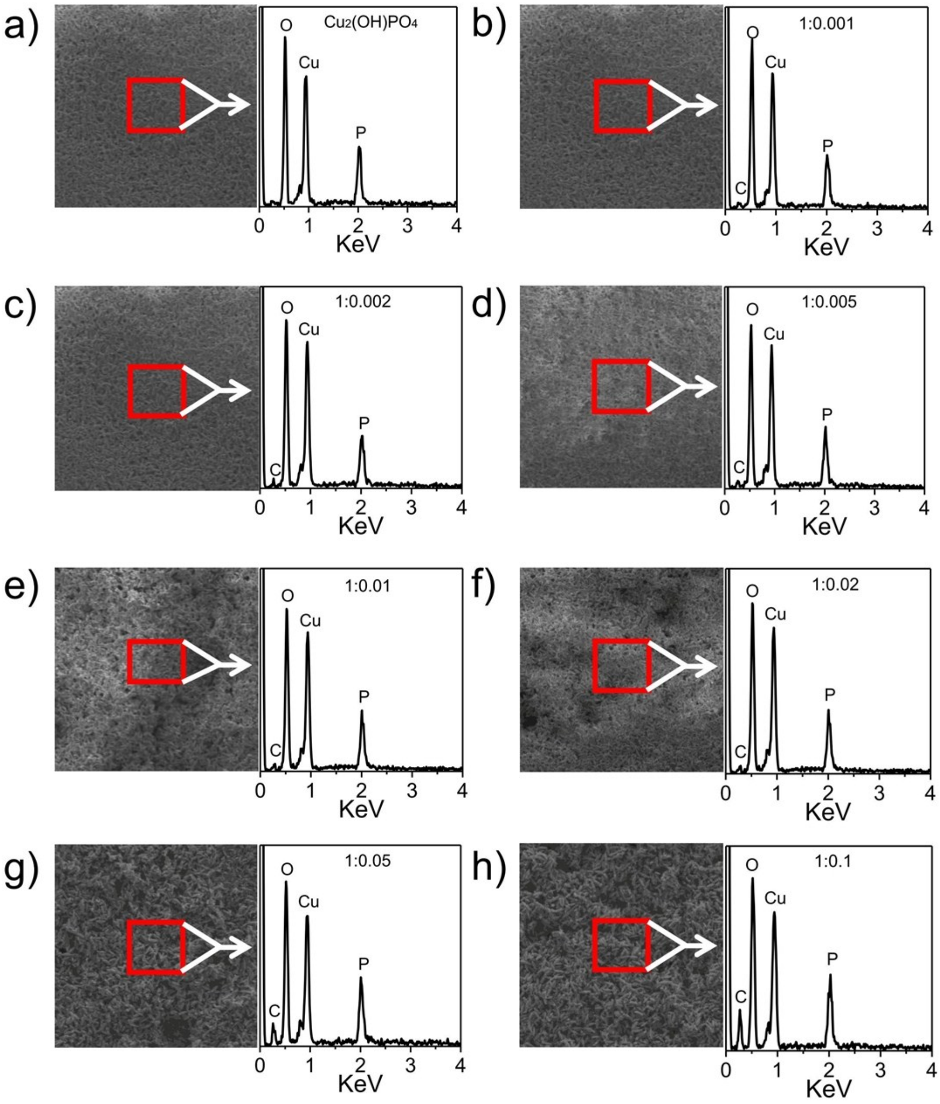

Fig. S6 EDS spectra of the as-prepared samples of that pure $Cu_2(OH)PO_4$ (a), sample 1:0.001 (b), sample 1:0.002 (c), sample 1:0.005 (d), sample 1:0.01(e), sample 1:0.02 (f), sample 1:0.05 (g) and sample 1:0.1 (h).

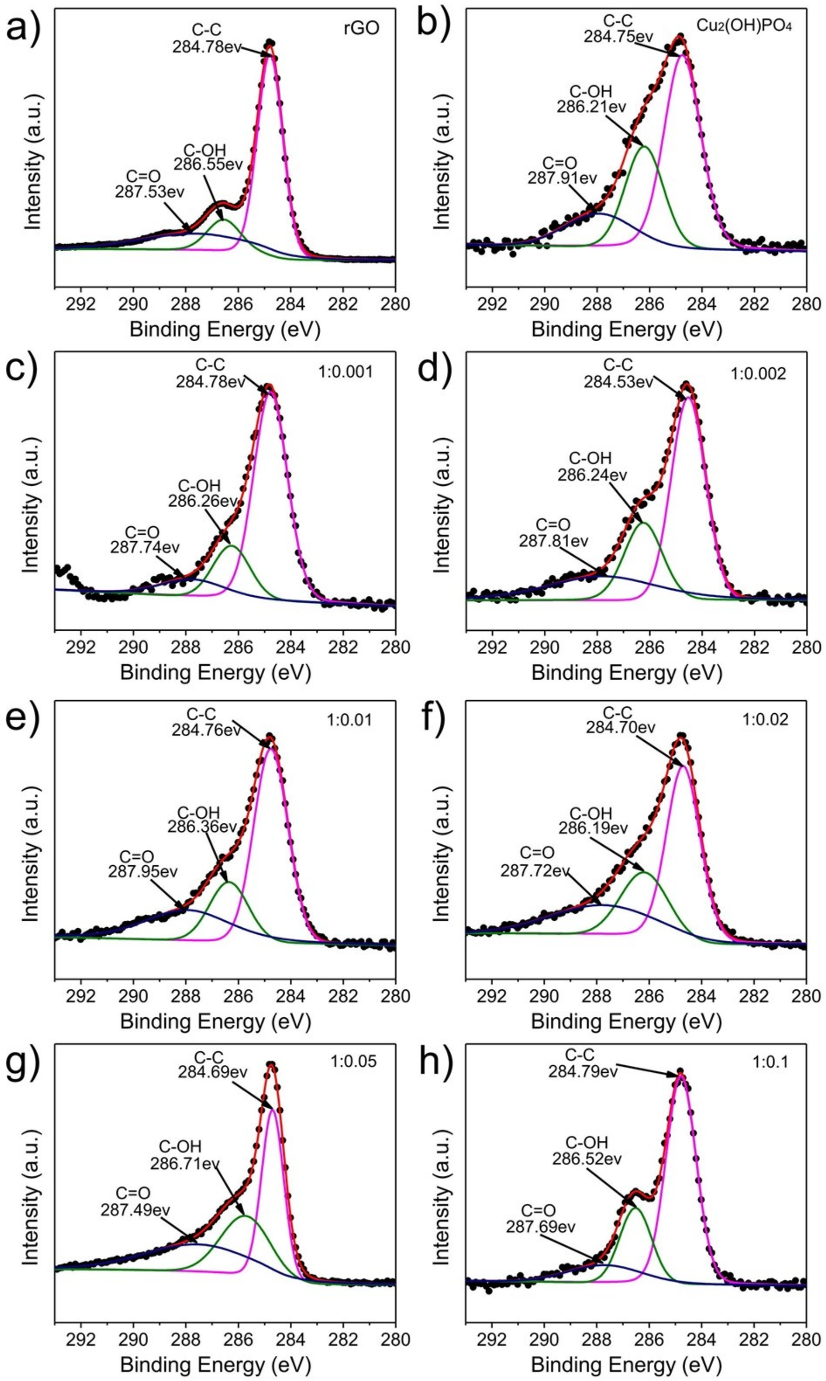

Fig. S7 C 1s XPS spectra of rGO (a), pure $Cu_2(OH)PO_4$ (b), sample 1:0.001 (c), sample 1:0.002 (d), sample 1:0.01 (e), sample 1:0.02 (f), sample 1:0.05 (g) and sample 1:0.1 (h).

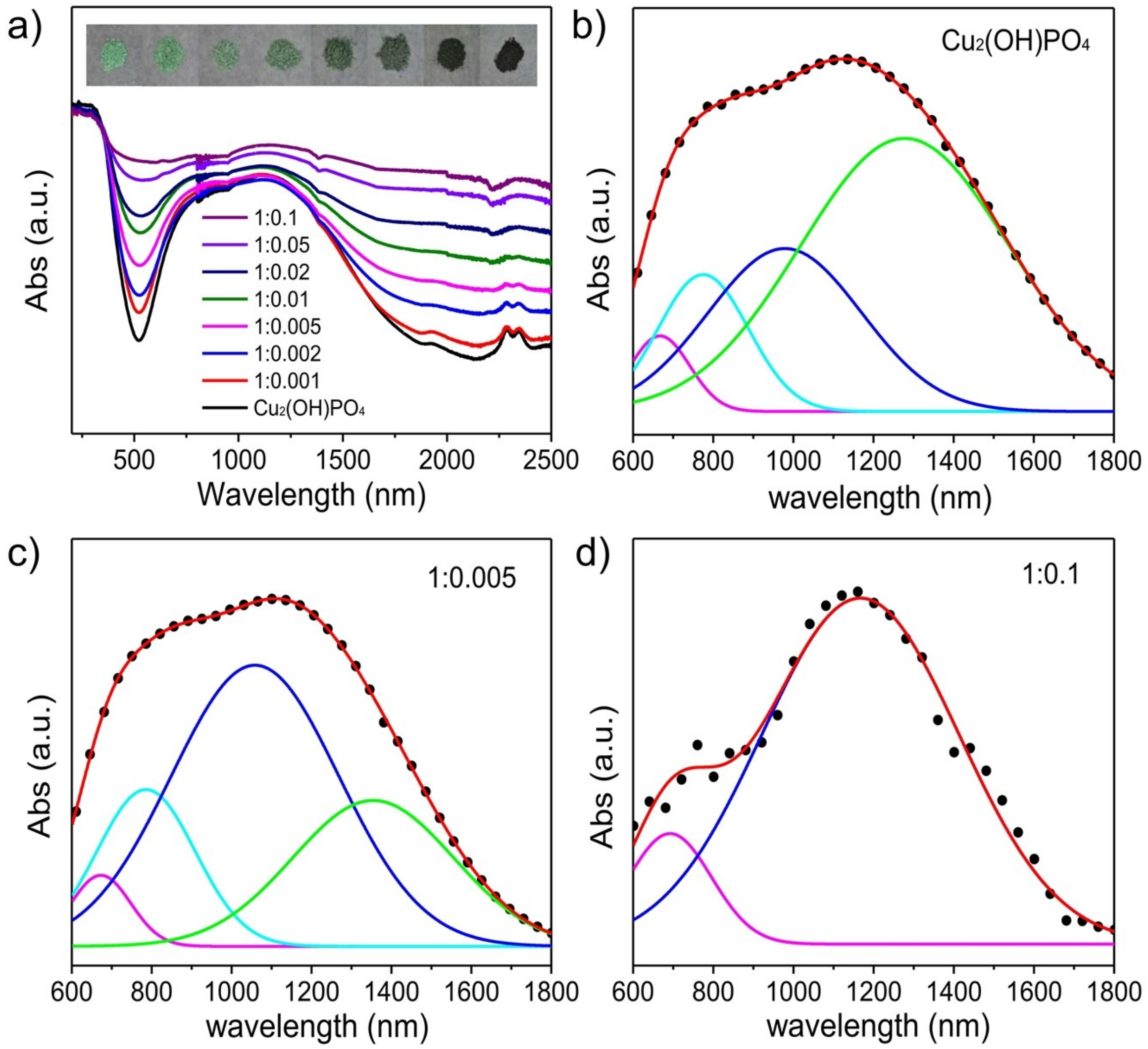

Fig. S8 (a) UV-vis-NIR absorption spectra of pure $Cu_2(OH)PO_4$ and $Cu_2(OH)PO_4$/rGO nanocomposites. Inset: Photographs of pure $Cu_2(OH)PO_4$ and $Cu_2(OH)PO_4$/rGO nanocomposites. (b) Enlarged UV-Vis-NIR absorption spectrum of pure $Cu_2(OH)PO_4$ ranges from 600 to 1800 nm, where infrared region is fitted with four Gaussian peaks: 1278 (Green), 978 (Blue), 775 (Oyan), and 667 nm (Magenta). c) Enlarged UV-Vis-NIR absorption spectrum of sample 1:0.005 ranges from 600 nm to 1800 nm, where infrared region is fitted with four Gaussian peaks: 1354 (Green), 1058 (Blue), 785 (Oyan), and 672 nm (Magenta). (d) Enlarged UV-Vis-NIR absorption spectrum of sample 1:0.1 ranges from 600 to 1800 nm, where the NIR region was fitted with two Gaussian peaks: 1167 (Blue) and 692 nm (Magenta).

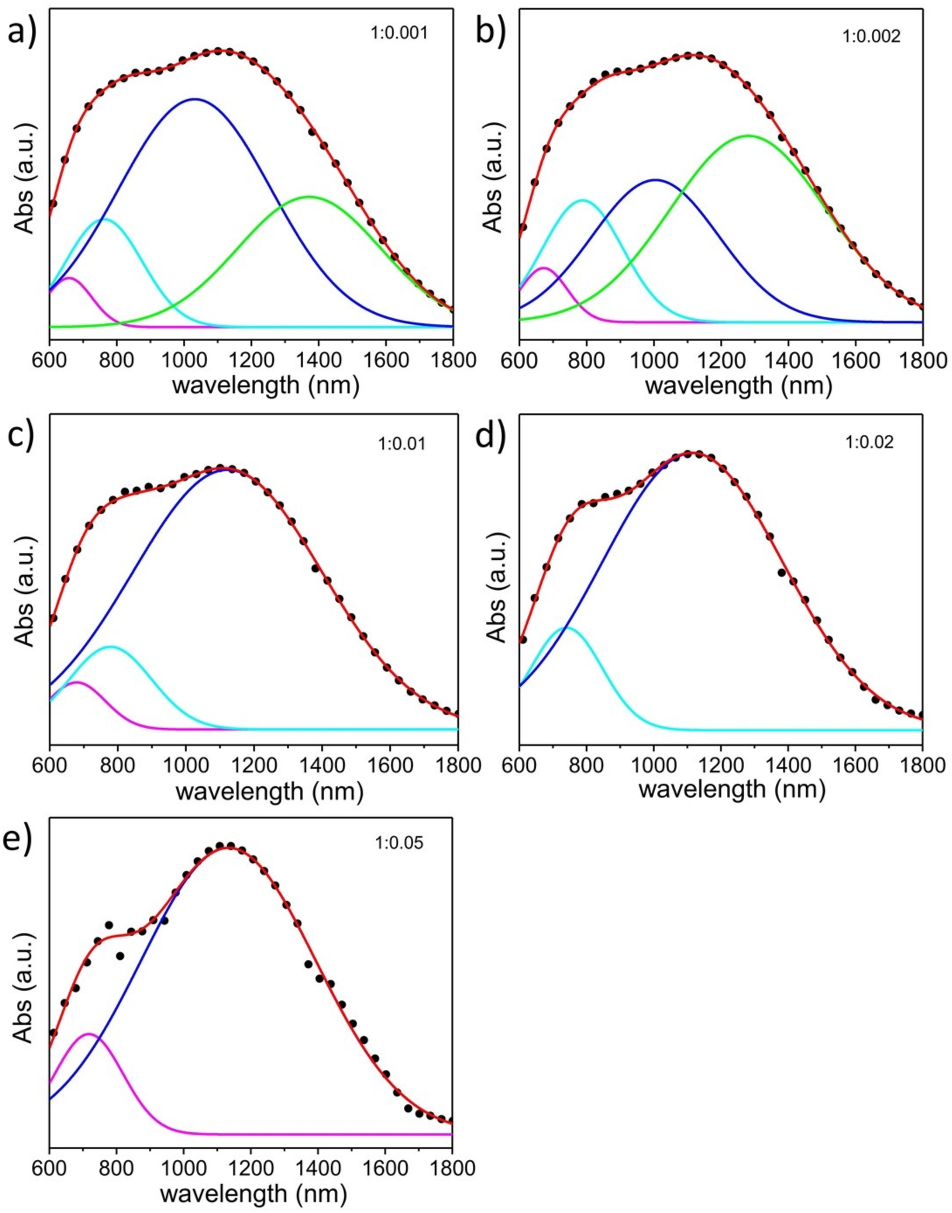

Fig. S9 (a) Enlarged UV-Vis-NIR absorption spectra of sample 1:0.001, where infrared region is fitted with four Gaussian peaks: 1371 (Green), 1031 (Blue), 762 (Oyan), 657 nm (Magenta). (b) Enlarged UV-vis-NIR absorption spectra of sample 1:0.002, where infrared region is fitted with four Gaussian peaks: 1280 (Green), 1004 (Blue), 788 (Oyan), 672 nm (Magenta). (c) Enlarged UV-vis-NIR absorption spectra of sample 1:0.01, where infrared region is fitted with four Gaussian peaks: 1122 (Blue), 778 (Oyan), and 679 nm (Magenta). (d) Enlarged UV-vis-NIR absorption spectra of sample 1:0.02, where infrared region is fitted with four Gaussian peaks: 1115 (Blue) and 740 nm (Magenta). (e) Enlarged UV-vis-NIR absorption spectra of sample 1:0.05, where infrared region is fitted with four Gaussian peaks: 1133 (Blue) and 717 nm (Magenta).

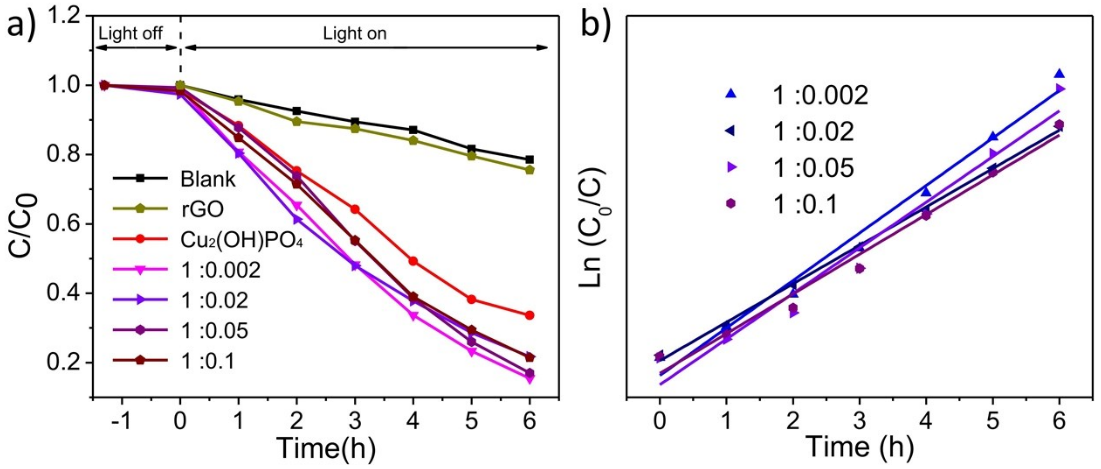

Fig. S10 (a) Photodegradation of 2,4-DCP over pure Cu2(OH)PO4 and $Cu_2(OH)PO_4$/rGO nanocomposites with 1:0.002, 1:0.02, 1:0.05 and 0.1 ratios with infrared light irradiation at 20-25 °C. (b) Plots of $Ln(C_0/C_t)$ versus time for $Cu_2(OH)PO_4$/rGO nanocomposites with 1:0.002, 1:0.02, 1:0.05 and 1:0.1 ratios.

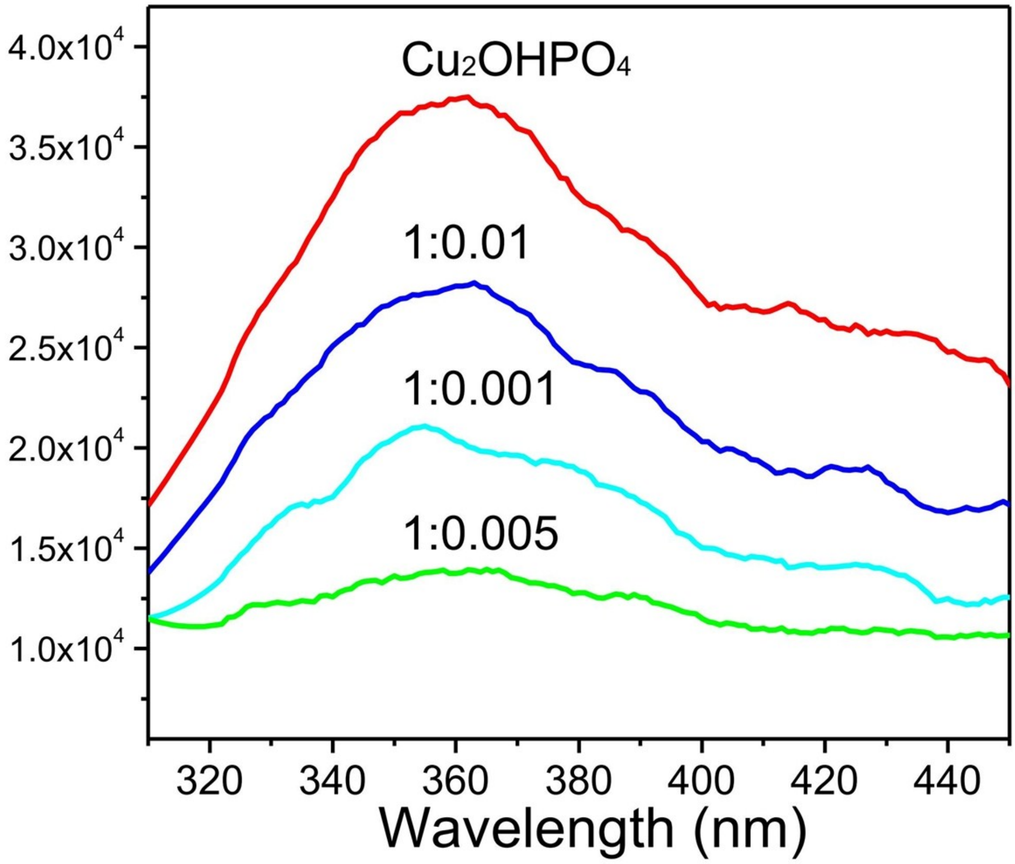

Fig. S11 Fluorescence spectra of $Cu_2(OH)PO_4$, 1:0.001, 1:0.005 and 1:0.1.

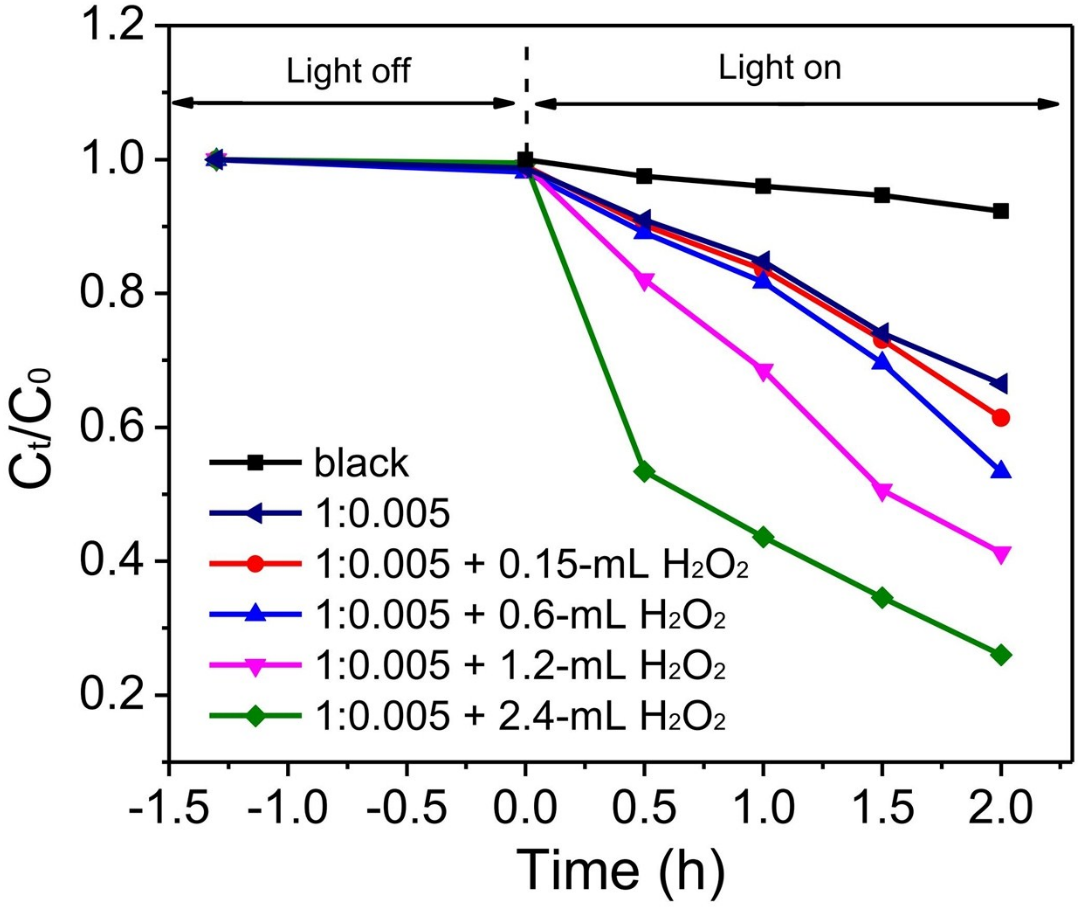

Fig. S12 Photodegradation efficiency of 2,4-DCP for sample 1:0.005 with the increase of the amount of H_2O_2 under infrared light irradiation.

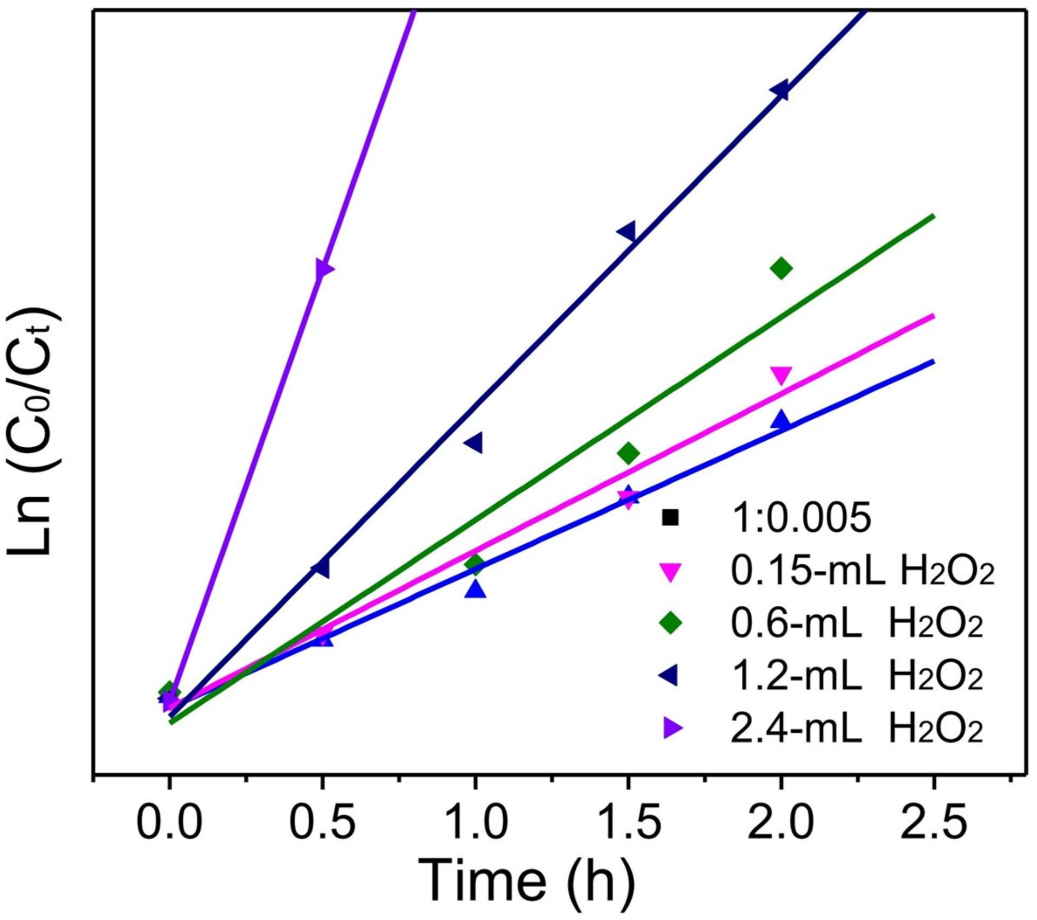

Fig. S13 Plots of Ln(C_0/C_t) versus time for sample 1:0.005 in the presence of different concentrations of H_2O_2.

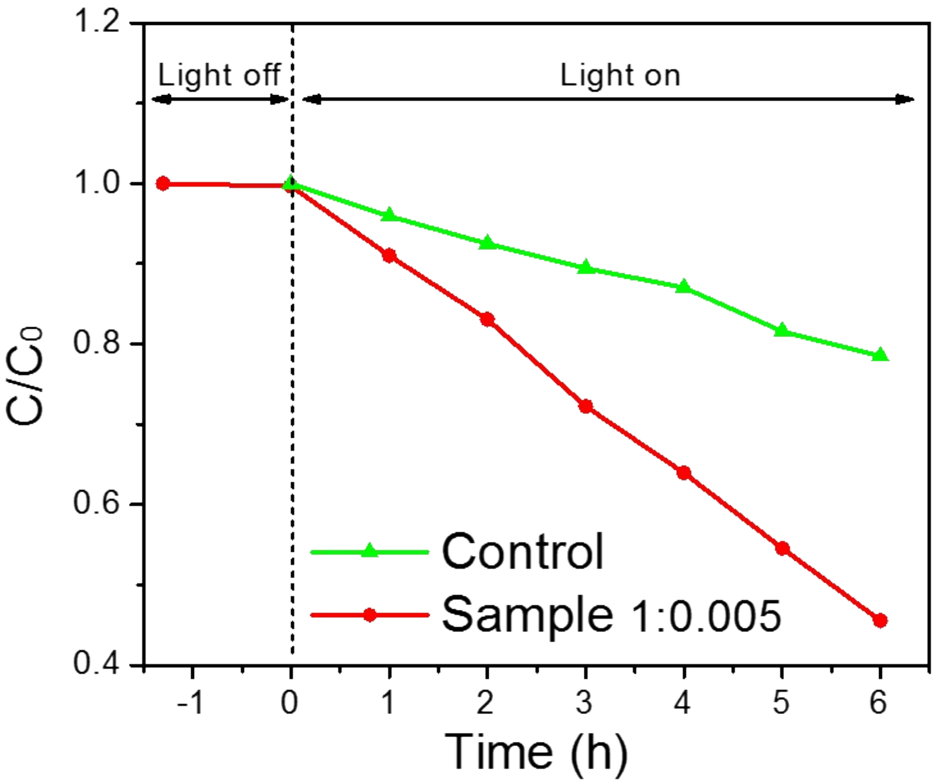

Fig. S14 Photodegradation of 2,4-DCP over pure $Cu_2(OH)PO_4$ and sample 1:0.005 with visible light irradiation at 20-25 °C.

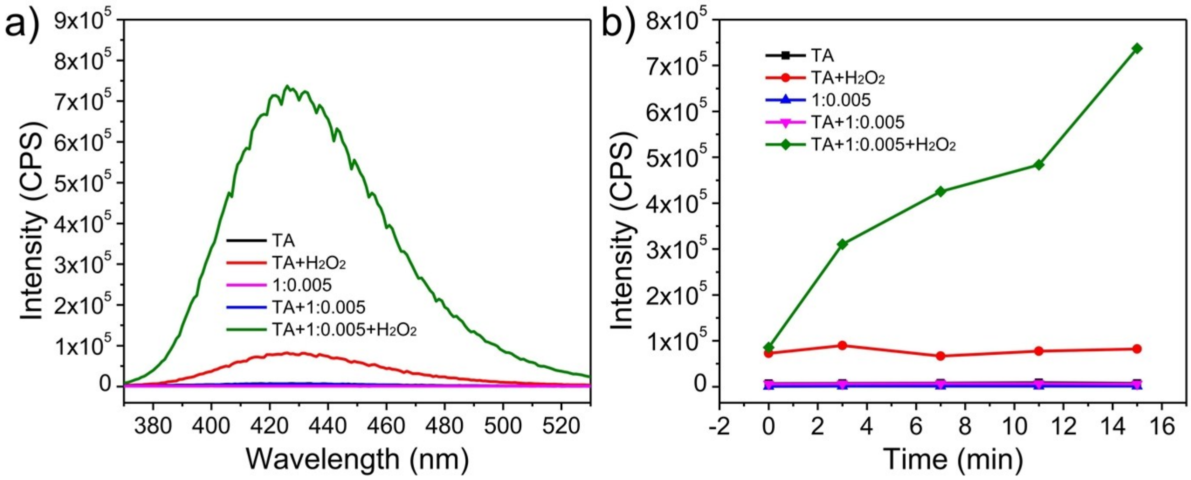

Fig. S15 (a) The HO˙ generation by sample 1:0.005 after 15min of infrared light irradiation. (b) The HO˙ generation over different treatments with infrared light irradiation as a function of irradiation time.

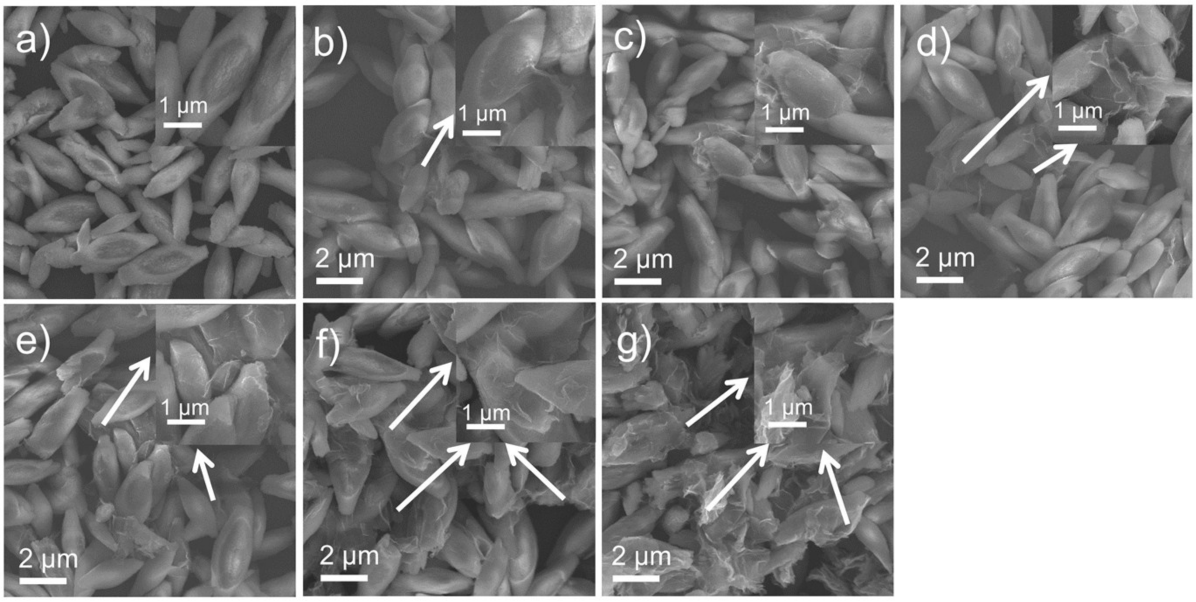

Fig. S16 SEM images of pure $Cu_2(OH)PO_4$ (a) and $Cu_2(OH)PO_4$/rGO nanocomposites with 1:0.001 (b), 1:0.002 (c), 1:0.01 (d), 1:0.02 (e), 1:0.05 (f) and 1:0.1 (g) ratios after repeated photocatalytic cycling. Inset: Enlarged SEM images of pure $Cu_2(OH)PO_4$ (a) and $Cu_2(OH)PO_4$/rGO nanocomposites.

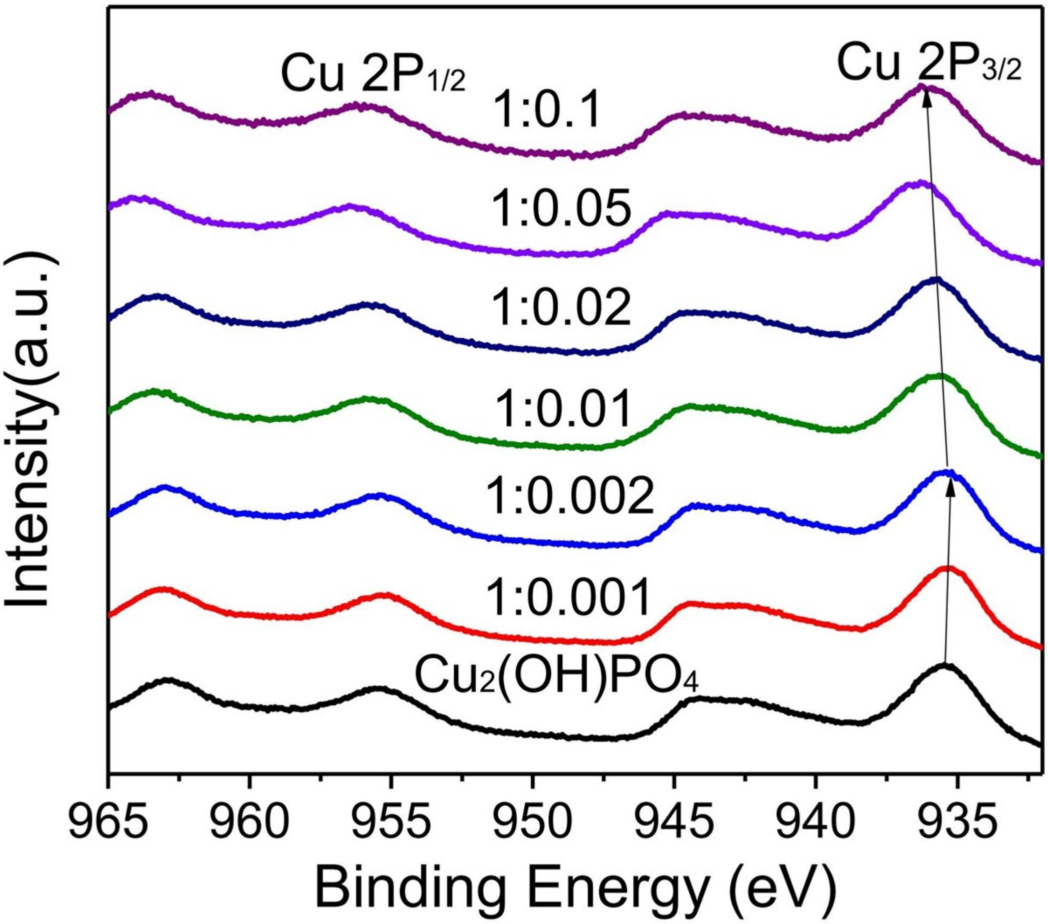

Fig. S17 Cu2p XPS spectra of pure $Cu_2(OH)PO_4$ and $Cu_2(OH)PO_4$/rGO nanocomposites after repeated photocatalytic cycling.

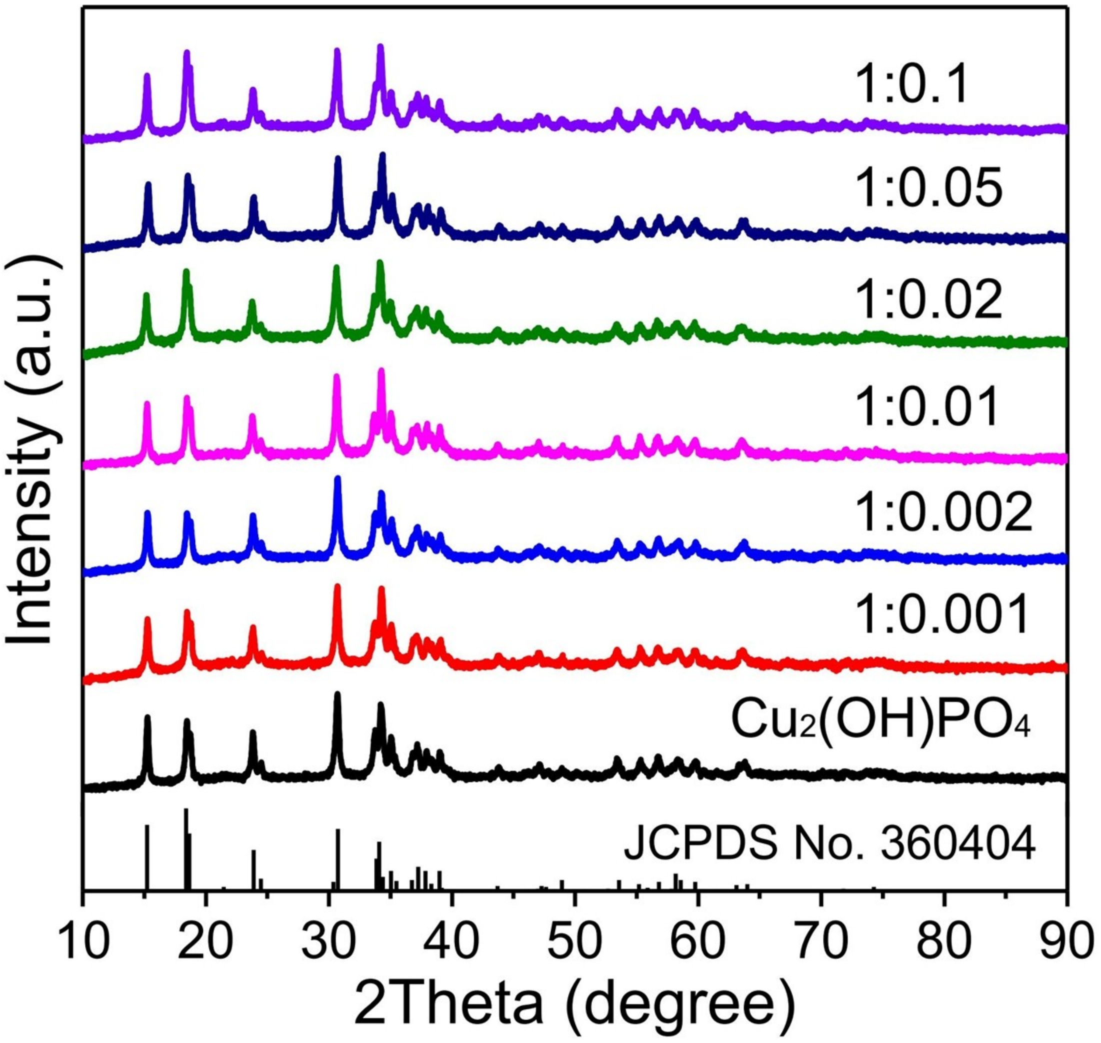

Fig. S18 XRD patterns of pure $Cu_2(OH)PO_4$ and $Cu_2(OH)PO_4$/rGO nanocomposites after repeated photocatalytic cycling.

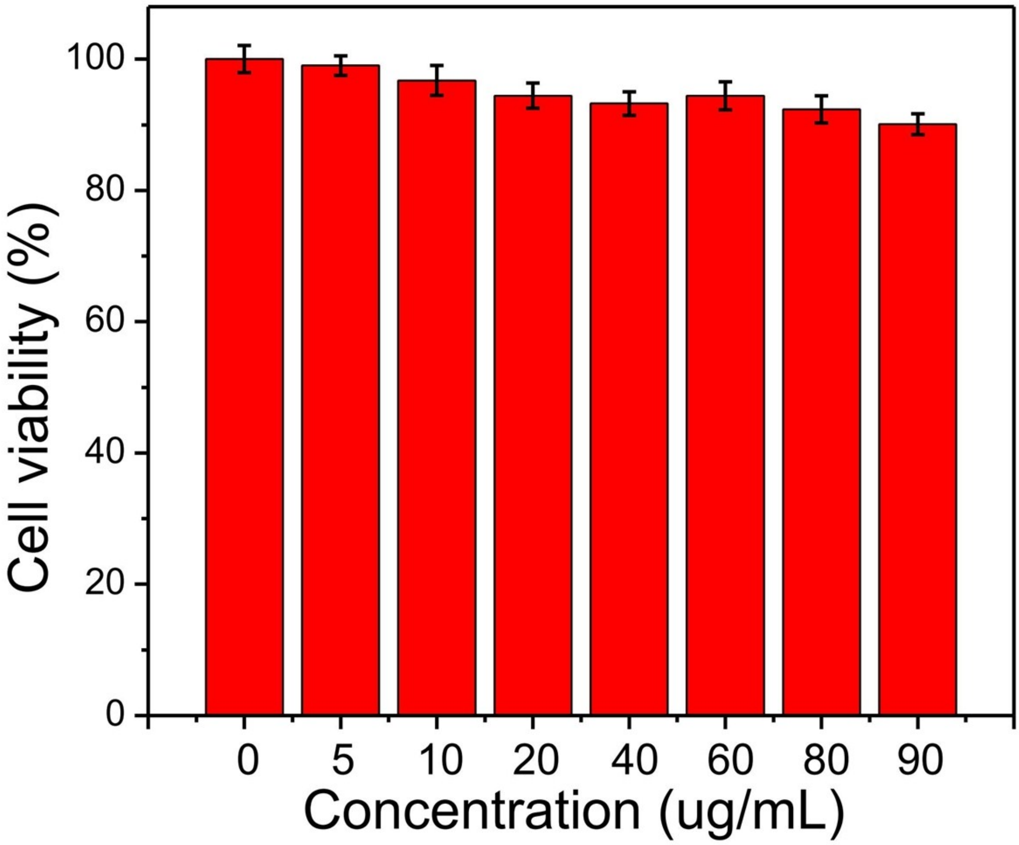

Fig. S19 Cytotoxicity of different concentrations of sample 1:0.005 to HUVECs (human umbilical vein endothelial cells).

Table S1. The reaction rate constant K of $Cu_2(OH)PO_4$/rGO nanocomposites.

**Sample**	***k* (h^{-1})**
$Cu_2(OH)PO_4$	0.190
1:0.001	0.264
1:0.002	0.311
1:0.005	0.326
1:0.01	0.246
1:0.02	0.251
1:0.05	0.298
1:0.1	0.260

References

1. W. S. Hummers, R. E. Offeman, J. Am. Chem. Soc. 1958, 80, 1339.

2. L. Yan, Y.-N. Chang, L. Zhao, Z. Gu, X. Liu, G. Tian, L. Zhou, W. Ren, S. Jin, W. Yin, H. Chang, G. Xing, X. Gao, Y. Zhao, Carbon 2013, 57, 120-129.
